# Genome-centric metatranscriptomes and ecological roles of the active microbial populations during cellulosic biomass anaerobic digestion

**DOI:** 10.1186/s13068-018-1121-0

**Published:** 2018-04-23

**Authors:** Yangyang Jia, Siu-Kin Ng, Hongyuan Lu, Mingwei Cai, Patrick K. H. Lee

**Affiliations:** B5423-AC1, School of Energy and Environment, City University of Hong Kong, Tat Chee Avenue, Kowloon, Hong Kong

**Keywords:** Anaerobic digestion, Metagenomics, Metatranscriptomics, Ecological roles, Lignocellulosic biomass

## Abstract

**Background:**

Although anaerobic digestion for biogas production is used worldwide in treatment processes to recover energy from carbon-rich waste such as cellulosic biomass, the activities and interactions among the microbial populations that perform anaerobic digestion deserve further investigations, especially at the population genome level. To understand the cellulosic biomass-degrading potentials in two full-scale digesters, this study examined five methanogenic enrichment cultures derived from the digesters that anaerobically digested cellulose or xylan for more than 2 years under 35 or 55 °C conditions.

**Results:**

Metagenomics and metatranscriptomics were used to capture the active microbial populations in each enrichment culture and reconstruct their meta-metabolic network and ecological roles. 107 population genomes were reconstructed from the five enrichment cultures using a differential coverage binning approach, of which only a subset was highly transcribed in the metatranscriptomes. Phylogenetic and functional convergence of communities by enrichment condition and phase of fermentation was observed for the highly transcribed populations in the metatranscriptomes. In the 35 °C cultures grown on cellulose, *Clostridium cellulolyticum*-related and *Ruminococcus*-related bacteria were identified as major hydrolyzers and primary fermenters in the early growth phase, while *Clostridium leptum*-related bacteria were major secondary fermenters and potential fatty acid scavengers in the late growth phase. While the meta-metabolism and trophic roles of the cultures were similar, the bacterial populations performing each function were distinct between the enrichment conditions.

**Conclusions:**

Overall, a population genome-centric view of the meta-metabolism and functional roles of key active players in anaerobic digestion of cellulosic biomass was obtained. This study represents a major step forward towards understanding the microbial functions and interactions at population genome level during the microbial conversion of lignocellulosic biomass to methane. The knowledge of this study can facilitate development of potential biomarkers and rational design of the microbiome in anaerobic digesters.

**Electronic supplementary material:**

The online version of this article (10.1186/s13068-018-1121-0) contains supplementary material, which is available to authorized users.

## Background

Anaerobic digestion (AD) is one of the most widely used methods to process waste to extract energy in the form of methane (CH_4_). Methanation of the carbon fraction of waste in AD is typically accomplished by a microbial consortium with at least three functional groups, namely fermenters, syntrophs and methanogens [1], that synergistically metabolize organic substrates to CH_4_ and carbon dioxide (CO_2_).

Although a wide range of microbes have been identified in AD operated under different conditions [2], we know relatively little about the activity of them, especially at genome level. A detailed understanding of the active microbes in AD is vital to improve the performance and control of AD systems. Many studies have analyzed phylogenetic marker genes (e.g., 16S rRNA gene) in AD [3–5], focusing only on the taxonomic composition of communities and extrapolating metabolic roles [6, 7]. Metagenomic sequencing with genome binning has made it possible to reconstruct genomes, which not only provide a more accurate phylogenetic profile than 16S rRNA gene-based studies [8], but also facilitate more reliable assignment of metabolic functions to specific microbial populations [9–14]. However, genome abundance alone does not necessarily reflect microbial activity, and the presence of genes also does not inform when or whether they are expressed. Sequencing of transcripts can provide insight into microbial activity and gene expression [15]. Due to previous limited RNA-sequencing depth, the large fraction of rRNA in metatranscriptomic datasets [16, 17] and the lack of appropriate reference databases for transcripts alignment and identification [18], genome-centric annotation of metatranscriptomes has only recently become possible. However, recent metatranscriptomic reports tend to focus only on a specific step of AD (e.g., carbohydrate hydrolysis [19] or long-chain fatty acid supplementation [20]). Hence, a detailed genome-centric view of the full AD meta-metabolic activity is still lacking.

Cellulosic biomass represents the most abundant carbon source for energy production in AD, including agricultural wastes and dedicates energy crops [21]. Genome-centric studies at the DNA level have been conducted to examine the microbiome during AD of cellulose [10, 22], but a genome-centric view of the functionally active populations is lacking. In this study, metagenomic and metatranscriptomic sequencing were applied to reconstruct the meta-metabolism of the active microbial community in five AD enrichment cultures digesting cellulose or xylan, the two major components of cellulosic biomass [23]. We previously assembled and enriched these communities at 35 or 55 °C using inocula from two full-scale wastewater anaerobic digesters [24]. Although the two digesters were not treating cellulosic waste, cellulose- and xylan-digesting functions could be enriched. Our previous 16S rRNA gene amplicon sequencing investigation found that the community diversity decreased and enrichment conditions drove the convergence of community structure along the 2 years enrichment period [24]. In this study, 107 population genomes (PGs) were reconstructed from the five enriched cultures via co-assembly of pooled metagenomes and differential coverage binning of each enrichment culture. The highly transcribed PGs in the metatranscriptomes of each culture were identified and their ecological roles and major meta-metabolic pathways were reconstructed and analyzed. Overall, this work provides a genome-centric view of the functionally active AD microbiome in different cultures during different phases of fermentation and offers insights that may be used to develop biomarkers for monitoring AD of cellulosic biomass to enable the operation of more efficient treatment and renewable energy systems.

## Methods

### Sample collection, metagenome and metatranscriptome library construction

**Fig. 1:**
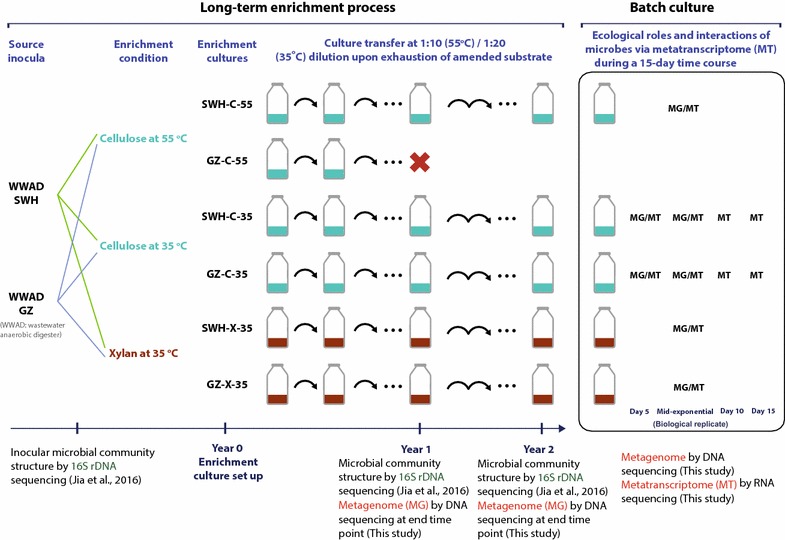
Schematic representation of the development of the enrichment cultures and sample collection for metagenomic and metatranscriptomic analyses

Metagenome and metatranscriptome samples were collected from five stable enrichment cultures previously established [24] with inocula collected from two full-scale anaerobic digesters (SWH, a municipal anaerobic digester treating waste activated sludge located at Shek Wu Hui, Hong Kong and GZ, an industrial anaerobic digester treating high-strength wastewater from a beverage manufacturing facility located at Guangzhou, China; both operated under mesophilic condition [25]). Briefly, cellulose or xylan (5 g/L) was used as the sole carbon and energy source and the enrichment cultures were incubated at 35 °C (cellulose or xylan) and 55 °C (cellulose only) for more than 2 years. Sub-culturing (1:10 or 1:20 dilution) was performed when the amended substrate was exhausted (approximately every 4 weeks) to select and enrich a community that could play a role in the AD of cellulose or xylan. Physiological properties including gas and volatile fatty acids production, substrate degradation and microbial communities of the enrichment cultures were monitored along the enrichment process, and stable profiles with no major changes were observed after 2 years of enrichment [24]. The five cultures were named GZ-C-35, SWH-C-35, GZ-X-35, SWH-X-35, and SWH-C-55 (or GC, SC, GX, SX, and S5 for short, respectively), where “C” denotes cellulose, “X” denotes xylan and “35” and “55” represent incubation temperatures of 35 and 55 °C, respectively. Samples for metagenomic sequencing were collected from the long-term enrichment cultures at the end of the first and second years (Fig. 1), denoted in the sample names by a suffix of “Y1” or “Y2”, respectively.

Short-term batch cultures were established from each enrichment culture after 2 years to obtain time course samples of community development over batch fermentation. Biological replicate cultures were set up for each enrichment culture. From both batch cultures grown on cellulose at 35 °C, samples were collected at days 5, 10, 15 and the mid-exponential growth phase (between days 5 and 10) (indicated by suffixes of “D5”, “D10”, “D15” and “Mid” respectively), while samples from both cultures grown on xylan at 35 °C and the culture grown on cellulose at 55 °C were collected at the mid-exponential growth phase only (Fig. 1). The mid-exponential growth phase was determined according to the physiological profiles of the enrichment cultures [24]. Metatranscriptomic and metagenomic sequencing were performed on batch culture samples as indicated in Fig. 1. Metagenomic sequencing was performed on all mid-exponential growth phase batch culture samples and on the day 5 samples of the batch cultures grown on cellulose at 35 °C to supplement the long-term metagenomes collected at the end of year 1 and 2 to facilitate differential coverage binning of PGs from each enrichment culture (Fig. 1). Time course experiments and 55 °C were only tested with cellulose to demonstrate the temporal transcription patterns and how temperature influenced the community.

Culturing, analytical measurements, sample collection, and genomic DNA extraction were performed as previously described [24]. RNA extraction, removal of genomic DNA, RNA quality assessment, and cDNA synthesis were performed as previously described [26]. DNA or RNA was extracted from each of the biological replicate cultures and then pooled by equal mass into a single sample for sequencing. Ribosomal RNA was removed from extracted total RNA using the Ilumina Ribozero kit following the manufacturer’s instructions before library construction (resulting in only ~ 5% of metatranscriptomic reads in each sample were identified as rRNA). A total of 16 metagenomic libraries and 11 metatranscriptomic libraries were sequenced (Fig. 1 and Additional file 1) on an Illumina HiSeq 4000 platform following the manufacturer’s instructions.

### Co-assembly, annotation and binning of metagenomes

FASTX-Toolkit (http://hannonlab.cshl.edu/fastx_toolkit/) and Trimmomatic v0.35 [27] were used to trim, filter (LEADING:3 TRAILING:3 SLUDUBGWINDOW:4:800 MINLEN:50) and check the quality of the metagenomic reads. De novo co-assembly was performed for each of the five enrichment cultures (Additional file 2). Specifically, pooled metagenomic clean reads from different time points of the same enrichment culture (Additional file 2) were co-assembled using IDBA-UD [28] with a maximum k-mer size of 100 and a minimum contig length of 800 bp. Open Reading Frames (ORFs) of the assembled contigs were predicted using Prodigal v2.60 [29] in metagenome mode. Putative protein functions were obtained by mapping ORFs to the SEED (release 70) [30], COG (2003-205) [31] and NCBI non-redundant (retrieved 26 July 2016) databases using DIAMOND [32] with a minimum identity of 40%. ORF KEGG annotations were obtained by submitting ORF sequences to the KEGG Automatic Annotation Server [33]. Carbohydrate-active enzymes (CAZymes) [34] and the putative protein domains were identified by searching the ORFs against the hidden Markov model (HMM) profiles of CAZy family domains [35] and Pfam (30.0) [36] using HMMER [37]. Sequences with inconsistent CAZyme domain annotation were identified and discarded by a customized pipeline. Reads of metagenomes at different time points from the same enrichment culture were mapped to the co-assembled contigs of their pan-metagenome, generating differential coverages, which were used for constructing PGs [38] and estimating the coverage of PGs in the respective metagenome. The extracted PGs from each enrichment culture were named according to the nomenclature of “enrichment culture-bin number”. For example, “GC.Bin1” denotes the first PG extracted from the GZ-C-35 enrichment culture. PGs were assessed for completeness and contamination based on the presence of lineage-specific, conserved, single-copy marker genes using the automated PG evaluation tool CheckM [39]. CheckM calculates completeness based on the number of expected marker genes present in a given PG and contamination based on the number of marker genes present in multiple copies. The relative abundance of a PG within a metagenome was obtained by averaging coverages of all contigs of that PG and dividing by the total number of reads in the metagenome. Phylogenetic analysis of the reconstructed PGs was carried out using PhyloPhlAn [8] and the online tool MiGA [40]. PhyloPhlAn uses four hundred conserved proteins from more than 3700 finished and draft microbial genomes for the construction of high-resolution phylogenetic tree, while MiGA assigns taxonomic classification based on the maximum Average Amino Acid Identity (AAI) found against the 11,566 reference genomes in the NCBI_prok database (version 6 March 2018). The two phylogenetic classification methods gave consistent taxonomic assignment for majority of the PGs (Additional file 4) and the PhyloPhlAn results were adopted for subsequent analyses. The genome-wide Average Nucleotide Identity (gANI) of the PGs against their neighboring reference genome was calculated using the JGI ANI calculator [41] (Additional file 4). The phylogenetic tree based on PhyloPhlAn results was drawn with GGTREE [42]. The metabolism of each PG was manually curated using KEGG annotations and annotations from the other databases mentioned above as required.

### Evaluation of transcriptional profiles of population genomes

Paired-end metatranscriptomic reads were trimmed and filtered with the same procedure and parameters as the metagenomic datasets, followed by removal of reads derived from non-coding RNA sequences (including rRNA) as defined in the rfam database [43, 44] using blastn [45]. RSEM [46] was used to quantify the transcriptional abundances of ORFs predicted from the PGs as well as the unbinned contigs, expressed as transcripts per million (TPM) of the total RNA reads in a sample [47]. Transcript abundances for functional categories were obtained by summing the transcriptional abundances of all ORFs assigned to a category. The quantitative level of transcription of a PG at each time point was determined by summing the TPM values of all ORFs assigned to that PG.

## Results

### Sequencing, co-assembly and binning

Sixteen metagenomic libraries were sequenced from samples of the long-term enrichment cultures and short-term batch cultures (Fig. 1). 848.5 million (17.9–83.7 million per sample) high-quality metagenomic reads were retained following quality control (Additional file 1a). Eleven metatranscriptomic libraries were sequenced from the short-term batch cultures (Fig. 1), yielding 617.0 million (35.4–71.3 million per sample) reads after quality control (Additional file 1b).

To assemble PGs from each enrichment culture, reads from different metagenomes of the same enrichment culture (Additional file 2) were pooled to form a pan-metagenome and de novo co-assembly was performed on each pan-metagenome. From the five pan-metagenomes, 70,207 contigs (523 Mbp) were generated after co-assembly, ranging in length from 800 bp–1.1 Mbp, with a mean N50 of 34,370 bp (17,716–68,909 bp per pan-metagenome). More than 90% of reads in each enrichment culture were assembled (Additional file 1a). Detailed co-assembly statistics are provided in Additional file 2.

Contigs from each pan-metagenome were binned by differential coverage to reconstruct PGs (Additional file 3). 107 PGs were reconstructed from the five pan-metagenomes (Additional file 2), accounting for 69–93% of reads in each metagenome (Additional file 1a). The estimated genome completeness of the PGs averaged > 90%, and the estimated contamination averaged 1.7% (Additional file 4). The PGs had an average of 2893 predicted ORFs (1186–6045 per PG) (Additional file 4). Details of the PGs are provided in Additional file 4.

Phylogenetic analysis based on PhyloPhlAn found that the 107 PGs spanned six known phyla plus an unknown archaeal phylum (GX.Bin23), with only three PGs having a gANI value greater than 96.5% and thus could be confidently classified to the species level [41] (Additional file 4). PGs were assigned to eight orders (*Anaerolineales*, *Bacteroidales*, *Clostridiales*, *Desulfovibrionales*, *Methanobacteriales*, *Selenomonadales*, *Spirochaetales*, and *Thermoanaerobacterales*), with *Methanobacteriales* (seven PGs) and *Clostridiales* (80 PGs) being the archaeal and bacterial orders with the most reconstructed PGs, respectively (Additional file 5).

### Transcriptional profiles of the PGs

**Fig. 2:**
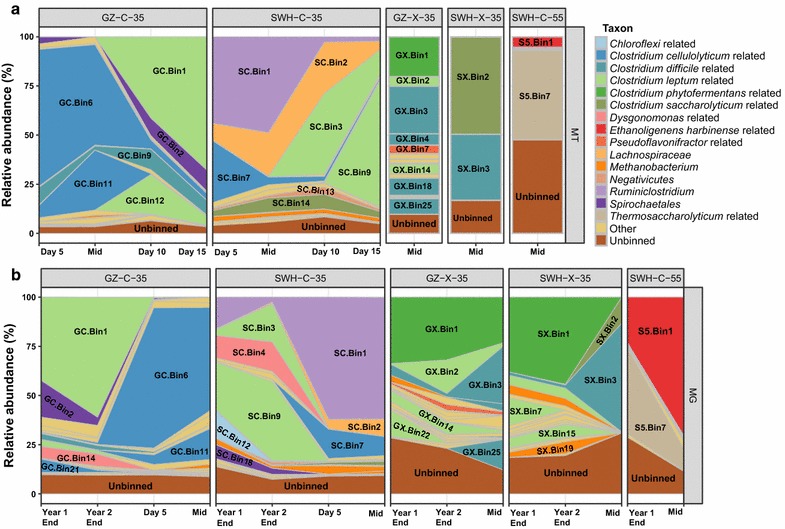
**a** Transcriptional level of the reconstructed PGs from each enrichment culture during batch experiments. The relative abundance was calculated based on the total sum of TPM values of all the ORFs of a PG divided by the sum of TPM values of all the ORFs of a culture at a particular time point. **b** Relative abundance of PGs in the metagenome of each enrichment culture along the enrichment process. The relative abundance was calculated based on the coverage of contigs of each PG within a metagenome. Only the highly transcribed or highly abundant PGs were labeled in each plot and the taxonomic classification of the PGs was based on their phylogenetic placement by PhyloPhlAn (see Fig. 3a)

**Fig. 3:**
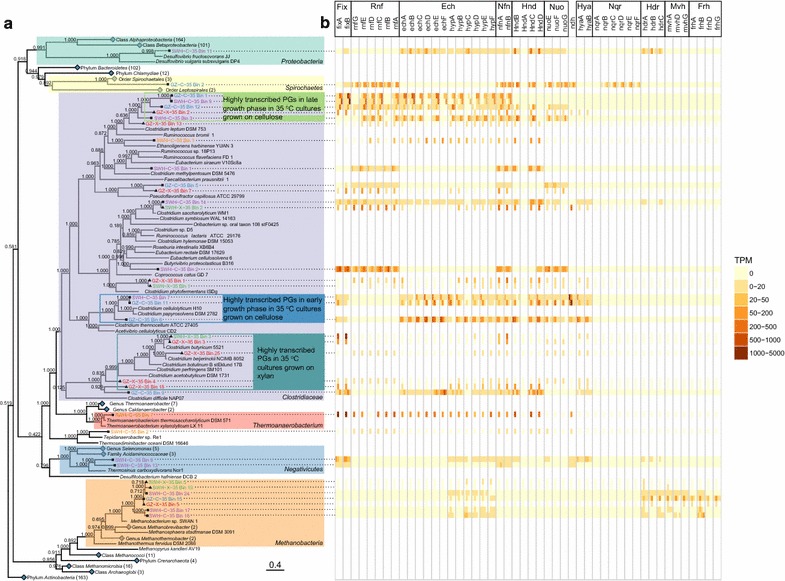
**a** Phylogenetic tree showing the placement of selected highly transcribed PGs. While 3737 reference genomes were used for alignment, only a subset of the aligned references was displayed. Blue diamonds indicate collapsed monophyletic clades with the number of reference genomes indicated in the brackets, black squares represent PGs from cultures amended with cellulose, and black triangles with xylan. Monophyletic clades of interest have been highlighted and bootstrap values (based on 100 iterations) are shown on internal nodes. The tree was midpoint-rooted. The scale bar indicates the evolutionary distance (substitution/site). PGs were color-coded by their enrichment culture, indigo for GZ-C-35, purple for SWH-C-35, red for GZ-X-35, green for SWH-X-35, and orange for SWH-C-55. **b** A heatmap showing the transcriptional level of the major energy conservation complexes and hydrogenases based on the TPM values of the corresponding genes. Time points in the order of day 5, mid-exponential growth phase, day 10 and day 15 were arranged as columns in the heatmap for each gene

Using RSEM, 32–66% of the metatranscriptomic reads were mapped to ORFs predicted from their source metagenomes (Additional file 1b). Of the 107 reconstructed PGs, only a subset was considered highly transcribed in the metatranscriptomes based on TPM values and the abundant PGs in the DNA were also the ones with high transcription in most of the communities (Fig. 2). Although all the enrichment cultures produced CH_4_ (1–1.5 mM from the 35 °C cultures, and < 0.2 mM from the 55 °C cultures after 0.5 g of substrate was consumed) [24], methanogen-related PGs were not highly transcribed in the 35 °C cultures, and no methanogen PG was reconstructed from the 55 °C culture. Large shifts in the highly transcribed populations were observed in both 35 °C cultures grown on cellulose during the batch fermentation, with *Clostridium cellulolyticum*-related PGs (GC.Bin6, GC.Bin11 in GZ-C-35; SC.Bin7 in SWH-C-35) and *Ruminococcus*-related PGs (SC.Bin1 in SWH-C-35) highly transcribed at day 5 and in the mid-exponential growth phase, while *Clostridium leptum*-related PGs (GC.Bin1, GC.Bin12 in GZ-C-35; SC.Bin3, SC.Bin9 in SWH-C-35) were more highly transcribed at days 10 and 15. Transcripts from PGs that clustered phylogenetically with *C. cellulolyticum* dominated the metatranscriptomes in the early growth phase during fermentation, while others that clustered with *C. leptum* dominated the late growth phase metatranscriptomes (Fig. 3a). However, the constitutively transcribed PGs in both 35 °C cultures grown on cellulose were different and scattered across the phylogenetic tree, including PGs related to the order *Spirochaetales* (GC.Bin2) and genus *Clostridium* (GC.Bin9) in GZ-C-35, and related to species *Clostridium saccharolyticum* (SC.Bin14) and *Butyrivibrio proteoclasticus* (SC.Bin2) in SWH-C-35 (Figs. 2a , 3a).

The two 35 °C cultures grown on xylan had different transcription profiles in the mid-exponential growth phase, with SWH-X-35 dominated by two highly transcribed PGs while GZ-X-35 had a more diverse group of PGs that were found to be expressing transcripts (Fig. 2a). Several of the highly transcribed PGs (GX.Bin3, GX.Bin4, GX.Bin18, GX.Bin25 from GZ-X-35 and SX.Bin3 from SWH-X-35) from the cultures grown on xylan clustered phylogenetically, forming a clade branching deeply within the *Clostridiales* (Figs. 2a, 3a). PGs related to *Clostridium butyricum* (GX.Bin3, SX.Bin3) and *Clostridium beijerinckii* (GX.Bin25) were included in this clade. *Clostridium phytofermentans*-related PGs (GX.Bin1, SX.Bin1) were also detected in both 35 °C cultures grown on xylan, though GX.Bin1 was abundant in the metagenome and highly expressing transcripts in the metatranscriptome in the GZ-X-35 culture, SX.Bin1 was abundant in the metagenome (at the end time points), but not expressing many transcripts in the metatranscriptome in the SWH-X-35 culture. One *C. saccharolyticum*-related PG (SX.Bin2) was also highly transcribed in the SWH-X-35 culture at mid-exponential growth phase (Fig. 2a).

Highly transcribed PGs in the 55 °C culture grown on cellulose (SWH-C-55) included PGs related to *Ethanoligenens harbinense* (S5.Bin1) and *Thermoanaerobacterium thermosaccharolyticum* (S5.Bin7) (Figs. 2a, 3a). S5.Bin1 dominated the metagenome of SWH-C-55 (~ 70% metagenome relative abundance) at mid-exponential growth phase, but contributed a much lower fraction of transcripts (~ 5% metatranscriptome relative abundance) (Fig. 2). A large fraction of SWH-C-55 metatranscriptomic reads were mapped to unbinned contigs, likely due to limited differential coverage from the small number of samples resulting in insufficient binning for this culture (Additional file 1).

### Hydrolysis of organic substrates

As the sole carbon and energy source for the enrichment cultures was insoluble or semi-soluble polymer macromolecules (cellulose or xylan), hydrolysis to more labile sugars was an essential prerequisite for the methanation of these compounds. The most highly transcribed bacterial populations also contributed the most transcribed CAZymes (Additional file 6). Of the ten most transcribed glycoside hydrolase (GH) families across the five cultures (Additional file 6), GH5, GH48 and GH9 were cellulases; GH1 and GH3 possessed both β-glucosidase and β-xylosidase activities; GH11 was annotated as endo-β-1,4-xylanase, and the remainder was functionally versatile. Except for two *C. cellulolyticum*-related PGs that highly transcribed in the early growth phase (GC.Bin11, SC.Bin7), two *C. leptum*-related PGs in the late growth phase (GC.Bin1, SC.Bin9) from the 35 °C cultures grown on cellulose, and one *C. butyricum*-related PG (SX.Bin3) from the mid-exponential growth phase of the culture grown on xylan, the other PGs had relatively similar GH transcriptional profiles (Additional file 6).

Carbohydrate binding modules (CBM) and cellulosomes are the two most commonly used substrate binding strategies for anaerobic hydrolysis of carbohydrate polymers [48, 49]. Two *C. cellulolyticum*-related PGs (GC.Bin11, SC.Bin7) in the 35 °C cultures grown on cellulose highly transcribed cellulosome-related genes in the early growth phase, including those for cohesins, dockerins and proteins with S-layer homology (SLH) domains. SLH domain genes were also transcribed in the SWH-C-55 and GZ-X-35 cultures, but mostly from unbinned contigs (Additional file 6). Highly transcribed CBMs in the 35 °C cultures grown on cellulose, including CBM50, CBM32, CBM46 and CBM3, were mostly from the *C. cellulolyticum*-related PGs (GC.Bin6, GC.Bin11, SC.Bin7), while in the 55 °C culture grown on cellulose, CBM50 and CBM22 were highly transcribed from the *T. thermosaccharolyticum*-related S5.Bin7. Although CBM46 and CBM3 transcripts were also detected from the *C. leptum*-related GC.Bin1 and unbinned contigs from GZ-C-35, transcription of most CBMs declined over time during batch fermentation for both 35 °C cultures grown on cellulose. The most-transcribed CBM in the 35 °C cultures grown on xylan was CBM22, with the TPM value higher than 6400 from the *C. phytofermentans*-related GX.Bin1 in GZ-X-35 (Additional file 6). In SWH-X-35, the *C. butyricum*-related SX.Bin3 also transcribed other CBMs including CBM48 and CBM50 at moderate levels (Additional file 6).

Carbohydrate esterases and glycosyltransferases were transcribed by almost all the highly transcribed PGs in the five cultures (Additional file 6), consistent with their roles in substrate hydrolysis.

### Sugar and cation transporters

**Fig. 4:**
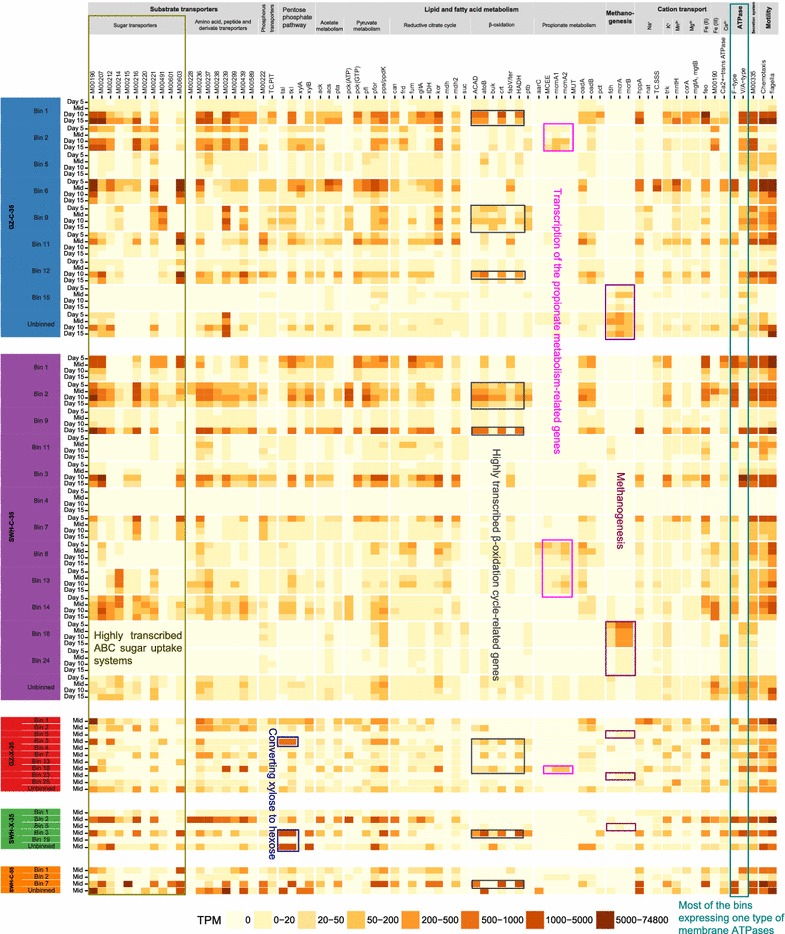
Heatmap showing the transcriptional profiles of major functions in AD process for the highly transcribed bacterial and methanogenic PGs. The PGs were organized by enrichment culture and color-coded as in Fig. 3. A few key functions are boxed and highlighted

For importing polysaccharide hydrolysis products, 11 ATP-binding cassette (ABC) transporters were highly transcribed across all cultures (Fig. 4). No relationship between culture condition or phase of fermentation and transporter transcript abundance was noted, except that M00215 (d-xylose transport system) and M00601 (putative chitobiose transport system) were exclusively highly transcribed in the 55 °C culture grown on cellulose by the *T. thermosaccharolyticum*-related S5.Bin7 (Fig. 4). Only two phosphotransferase system (PTS) modules (M00275 (cellobiose-specific II component) and M00276 (mannose-specific II component)) related to sugar import were highly transcribed. Cation transporters (sodium, potassium, magnesium, manganese, calcium, ferrous and ferric iron) were highly transcribed in all cultures (Fig. 4).

### Fermentation of glucose and xylose

Imported oligosaccharides are further hydrolyzed by intracellular cellulases or xylanases to glucose or xylose, respectively. While in the cellulose-amended cultures glucose could enter glycolysis directly, when xylose is the sole carbon source it must first be converted to hexose prior to entering glycolysis. Conversion of pentose to xylulose-5-phosphate and subsequently glyceraldehyde-3-phosphate, a glycolysis intermediate, can be achieved via the non-oxidative branch of the pentose phosphate pathway (PPP) or the phosphoketolase pathway (PKP) [50]. Genes for key PPP enzymes transketolase (Tkl) and transaldolase (Tal) were highly transcribed by the *C. butyricum*—(GX.Bin3, SX.Bin3), *C. phytofermentans*—(GX.Bin1) and *C. saccharolyticum*-related (SX.Bin2) PGs in the communities grown on xylan (Fig. 4), while the gene for the key PKP enzyme phosphoketolase (Xpk) was not highly transcribed in communities grown on xylan. With the exception of S5.Bin7, all highly transcribed PGs transcribed a complete set of genes for the Embden-Meyerhof-Parnas (EMP) glycolysis pathway and for pyruvate:ferredoxin oxidoreductase (PFOR), required to convert pyruvate generated from glycolysis to acetyl coenzyme A (CoA).

There are two pathways for AD communities to convert acetyl-CoA to acetate. In one pathway, acetyl-CoA is first converted to acetate-phosphate by phosphate acetyltransferase (Pta), and then acetate-phosphate is converted to acetate by acetate kinase (Ack), yielding ATP. In the other pathway, acetyl-CoA synthetase (Acs) converts acetyl-CoA to acetate [51]. The Pta-Ack pathway was the most highly transcribed from the PGs with high gene transcription, although the *acs* gene was also highly transcribed from the *C. cellulolyticum*-related GC.Bin6 in the early growth phase and *C. leptum*-related GC.Bin1 and SC.Bin9 in the late growth phase in the 35 °C cultures grown on cellulose (Fig. 4).

### Fatty acid metabolism

In AD, pyruvate can be converted to formate by pyruvate formate lyase (Pfl). The *pfl* gene was transcribed from several PGs in the 35 °C cultures, including PGs related to *C. cellulolyticum* (GC.Bin6, GC.Bin11, SC.Bin7), *C. leptum* (GC.Bin1, GC.Bin12, SC.Bin3, GX.Bin2), *Ruminococcus* (SC.Bin1), *C. butyricum* (GX.Bin3, SX.Bin3) and *C. saccharolyticum* (SX.Bin2) (Fig. 4). However, formate did not accumulate during fermentation [24], indicating it was consumed in the syntrophic transfer of electrons from fermenters to methanogens [52]. The *cdh* genes encoding the acetyl-CoA decarbonylase/synthase subunit of the acetyl-CoA synthase/carbon monoxide dehydrogenase (ACS/CODH) complex, the key enzyme complex in the Wood–Ljungdahl pathway (WLP), were negligibly transcribed across all cultures (< 23 TPM), even in several PGs carrying the *cdh* genes (GX.Bin4, S5.Bin2, S5.Bin4 and S5.Bin5). Together, these results suggest syntrophic acetate oxidation (SAO) via reverse WLP was not a major acetate metabolic pathway in these cultures. Most of the genes encoding the key enzymes in the alternative acetate oxidation pathway via the pyruvate–serine–glycine cleavage system [9, 13] were either absent in the PGs or negligibly transcribed, indicating insignificant acetate oxidation via this pathway. Genes for propionate metabolism via the methylmalonyl-CoA pathway and butanoic acid metabolism via the butyryl-CoA:acetate CoA-transferase (But) or butyrate kinase (Buk) were also negligibly transcribed (Fig. 4).

Genes encoding enzymes involved in β-oxidation including 3-hydroxyacyl-CoA dehydrogenase (HADH), enoyl-CoA hydratase (ECH), acyl-CoA dehydrogenase (ACAD) and electron-transfer-flavoprotein (FixA, FixB) were contiguously located (Additional file 7a) and consistently highly transcribed from PGs related to *C. leptum* (GC.Bin1, SC.Bin9, GC.Bin12, GX.Bin14), *B. proteoclasticus* (GC.Bin4, SC.Bin2), *C. butyricum* (GX.Bin3, SX.Bin3), *C. acetobutylicum* (GX.Bin18, GC.Bin9), and *T. thermosaccharolyticum* (S5.Bin7) (Figs. 3a, 4). Acetyl-CoA acetyltransferase (atoB) was found adjacent to the β-oxidation gene cluster in several PGs, including GC.Bin1, GC.Bin12, GX.B14, SC.Bin2 and SC.Bin9, and was consistently transcribed with genes involved in β-oxidation (Additional file 7a).

### Hydrogenesis and energy conservation

Anaerobic fermentation of sugars produces reduced protein ferredoxin (Fd_red_) and reduced nicotinamide adenine dinucleotide (NADH). Appropriate mechanisms and electron acceptors are needed to re-oxidize and recycle these reduced cofactors. In methanogenic systems, hydrogen- or formate-using methanogens can maintain low concentrations of hydrogen and formate (< 10 Pa and < 10 μM, respectively), making the use of protons as electron acceptors energetically feasible [53–55]. Hydrogenases were transcribed from almost all highly transcribed PGs (Fig. 3b), and hydrogenase subunits were contiguously encoded and consistently transcribed (Additional file 7). The highly transcribed energy converting [NiFe] hydrogenase (Ech) is a membrane-associated hydrogenase that oxidizes Fd_red_ to produce H_2_, simultaneously establishing a proton gradient [56, 57] that can drive oxidative phosphorylation to reclaim energy [58, 59].

Sodium gradients may also have contributed to the bacterial energy conservation strategy. The redox-driven membrane-bound sodium pump Rnf (*Rhodobacter* nitrogen fixation) complex operon was transcribed from many highly transcribed PGs (Fig. 3b and Additional file 7b). Reduced Fd is used by Rnf to drive simultaneous sodium export and NAD^+^ reduction. Another highly transcribed sodium pump was a membrane-bound pyrophosphatase, annotated by KEGG as a K^+^-stimulated pyrophosphate-energized sodium pump (HppA), which uses energy from pyrophosphate hydrolysis for sodium export (Fig. 4). Na^+^-transporting NADH:ubiquinone oxidoreductase (Nqr) was transcribed from two PGs, GC.Bin2 and SC.Bin18, both affiliated with the phylum *Spirochaetes* (Additional file 5). Na^+^-Nqr transfers electrons from NADH to ubiquinone and uses the free energy released by the redox reaction to export sodium [60]. Proton or sodium gradient-based membrane ATP synthase genes were transcribed from a broad range of PGs, with most transcribing only one type (*F* type or *V* type) (Fig. 4). Several PGs (e.g., GC.Bin6, SC.Bin1, GX.Bin1) highly transcribed the gene (TC.SSS) for solute:Na^+^ symporters, suggesting the sodium gradient could be used to drive substrate uptake (Fig. 4).

The NADH-dependent reduced ferredoxin:NADP(+) oxidoreductase (NfnAB) was moderately transcribed across many genomes, consistent with a previous study [59]. The NfnAB complex couples the exergonic reduction of NADP^+^ with reduced Fd and the endergonic reduction of NADP^+^ with NADH in a reversible reaction [61, 62], connecting NADH- and NADPH-specific metabolic reactions in another energy-conserving mechanism.

### Methanogenesis

Eight archaeal PGs were reconstructed from the four 35 °C cultures, ranging in relative abundance in the metagenomes from 0.01 to 6.1% (Fig. 2b), while no archaeal PG was reconstructed from the 55 °C culture grown on cellulose. Seven of the eight archaeal PGs were affiliated with the genus *Methanobacterium*, while one could not be classified further than the domain *Archaea* (Fig. 3a and Additional file 5). Genes encoding key hydrogenotrophic methanogenesis enzymes such as formate dehydrogenase (Fdh) and formylmethanofuran dehydrogenase (Fwd) were transcribed from methanogen PGs in the 35 °C cultures grown on cellulose, while detailed transcriptional profiles of methanogens from the cultures grown on xylan could not be determined due to low metatranscriptomic coverage (Fig. 4). No transcription of key acetoclastic methanogenesis genes including acetate kinase (Ack) and phosphotransacetylase (Pta) was detected from methanogen genomes. Although the overall metatranscriptomic coverage of the methanogen PGs was also relatively low (0.01–2.6%), the gene encoding methyl coenzyme M reductase (*mcrA*), which catalyzes the terminal step of methanogenesis, was relatively highly transcribed in the two 35 °C cultures grown on cellulose and moderately transcribed in the two 35 °C cultures grown on xylan, while it was not detected in the 55 °C culture grown on cellulose (Fig. 4).

## Discussion

### Taxonomic and functional convergence of the enrichment cultures

We previously reported a 16S rRNA gene-based analysis that found the five cultures analyzed in this study converged to a similar community structure when they were enriched under the same condition after 2 years, despite being established from different inocula [24]. Here, the reconstruction of PGs coupled with transcription profiles enabled us to identify the highly transcribed PGs in each community, infer the meta-metabolic networks and impute functional roles to the reconstructed PGs. Clear convergence, driven by incubation condition (substrate together with temperature), was found in both the phylogeny of the highly transcribed PGs and in their meta-metabolic roles.

In the two 35 °C cultures grown on cellulose, the highly transcribed PGs in the early growth phase (GC.Bin6, GC.Bin11, SC.Bin7) were phylogenetically close to *C. cellulolyticum* and *Clostridium papyrosolvens*, which are well documented for their cellulolytic capabilities, either by producing the super cellulolytic enzyme complex cellulosome [63–65] or multicomplex cellulase-xylanase systems [66, 67]. Cellulosome structural components including SLH, dockerin and cohesion were highly transcribed by GC.Bin6, GC.Bin11 and SC.Bin7 (Additional file 6), again suggesting the use of cellulosomes for cellulose hydrolysis. Different CBM families were preferred by different PGs, with GC.Bin6 highly transcribing CBM50 and CBM32, while GC.Bin11 and SC.Bin7 transcribed CBM46 and CBM3. This is consistent with the closer phylogenetic relationship between GC.Bin11 and SC.Bin7 than to GC.Bin6, and their more similar GH transcriptional profiles.

Another highly transcribed *Ruminococcus*-related PG (SC.Bin1) in the early growth phase in the SWH-C-35 culture was phylogenetic closely related to *Eubacterium siraeum* V10Sc8a, *Ruminococcus flavefaciens* FD1 and *Ruminococcus* sp. 18P13, all of which have been reported to have potential cellulolytic functions [68, 69], and close relatives of which have been detected in cellulolytic biogas reactors [70]. However, no cellulosome-related transcripts were detected in the SC.Bin1 transcriptome (Additional file 6), although *R. flavefaciens* has been reported to use cellulosomes to degrade cellulose in cow rumens [69], indicating phylogenetically close populations might still adopt different cellulose hydrolysis mechanisms.

Several highly transcribed PGs in the late growth phase of the 35 °C batch cultures grown on cellulose (GC.Bin1, GC.Bin12, SC.Bin9, SC.Bin3) were affiliated with *C. leptum* (Figs. 2a, 3a), which has been reported to be an abundant carbohydrate fermenter in the human gut, and to produce butyrate to support colonic mucosal function [64, 71]. However, the lack of transcription of key butyrate production genes (*buk*, *but*) from these PGs suggests they did not produce butyrate in our enrichment cultures. Furthermore, the highly transcribed β-oxidation pathway could efficiently scavenge any butyryl-related intermediates produced, preventing butyrate production and accumulation, which is consistent with the observation of negligible butyrate concentration during fermentation [24]. The lack of butyrate production in the enrichment cultures could be further explained by hydrogen- and formate-mediated syntrophic interactions between the fermenters and methanogens. The maintenance of a low hydrogen and formate concentrations by methanogens facilitates the use of protons as electron acceptors, resulting in an energetic preference for producing the more oxidized acetate rather than the more reduced butyrate [72]. However, the absence of syntrophic acetate oxidation bacteria (SAOBs) or acetoclastic methanogens led to the accumulation of acetate. The high transcription of acyl-CoA dehydrogenase-FixAB complex and other genes in the β-oxidation gene cluster (Additional file 7a) in the late growth phase in the batch cultures was likely involved in the scavenging of fatty acids from cell wall lipids and other cellular debris as the initial feedstock and glycolysis products became relatively scarce. Genes encoding for the entire fatty acid β-oxidation cycle have been found in the genomes of long-chain fatty acid degraders in a previous stable isotope probing study [73] and phylogenetically diverse bacterial groups have been identified to be active in methanogenic sludge degrading long-chain fatty acids in another stable isotope probing study [74]. High transcription of the membrane-bound and energy-conserving hydrogenase (Fig. 3b) and the active cooperation with hydrogenotrophic methanogens support the possibility of fatty acids consumption by these highly transcribed PGs in the late growth phase, due to the low hydrogen and formate concentrations environment maintained by hydrogenotrophic methanogens [52].

Unlike the cellulose-amended cultures, most of the highly transcribed PGs from the cultures grown on xylan formed a clade which includes *C. butyricum* (Fig. 3a), again demonstrating taxonomic convergence driven by enrichment condition. Similar transcriptional profiles of the sugar transporters between the cultures grown on cellulose and xylan are likely due to the non-specific substrate binding of the transporters. On the other hand, the most highly transcribed CBM22 in the cultures grown on xylan is annotated to have xylan-binding function [34]. Contrary to a previous ^13^C-based analysis finding that 40% xylose catabolism in *Clostridium acetobutylicum* is via PKP [50], xylose appears to have entered glycolysis solely via PPP in the two cultures grown on xylan (Fig. 4). Although several close relatives of these PGs including *C. butyricum* 5521, *C. beijerinckii* NCIMB8052, *C. acetobutylicum* DSM1731, *Clostridium botulinum* B str. Eklund 17B and *C. perfringens* SM101 have been reported as potential butyrate fermenters [75], only negligible transcription of the *buk*-encoding gene was observed. As in the 35 °C cultures grown on cellulose, the highly transcribed acyl-CoA dehydrogenase-FixAB complex and β-oxidation related gene cluster might be involved in scavenging fatty acids from the cell wall lipids and other cellular debris.

Most of the PGs reconstructed from the 55 °C cellulose enrichment culture were phylogenetically distant from those of the 35 °C cultures (Fig. 3a and Additional file 5), which is likely due to the long-term selective pressure under different temperatures. The most highly transcribed S5.Bin7 was closely related to *T. thermosaccharolyticum* DSM 571, whose thermophilic starch-hydrolyzing capability has been well documented [76]. However, only GH1 and GH109 from the top 10 GH categories were transcribed from S5.Bin7 (Additional file 6). As with the 35 °C cultures, S5.Bin7 in the 55 °C culture grown on cellulose might play the role of potential fatty acids scavenger as indicated by the highly transcribed acyl-CoA dehydrogenase-FixAB complex and β-oxidation related gene cluster (Fig. 4 and Additional file 7a). The relatively lower transcription of the *E. harbinense*-related PG S5.Bin1 (Fig. 2 and Additional file 5) may be because *E. harbinense* YUAN 3 has a preference for 35 °C [77]. However, S5.Bin1 transcribed a more versatile set of GHs compared to the *T. thermosaccharolyticum*-related S5.Bin7, indicating its possible role as the primary cellulose degrader (Additional file 6).

### Ecological roles of microbial populations

**Fig. 5:**
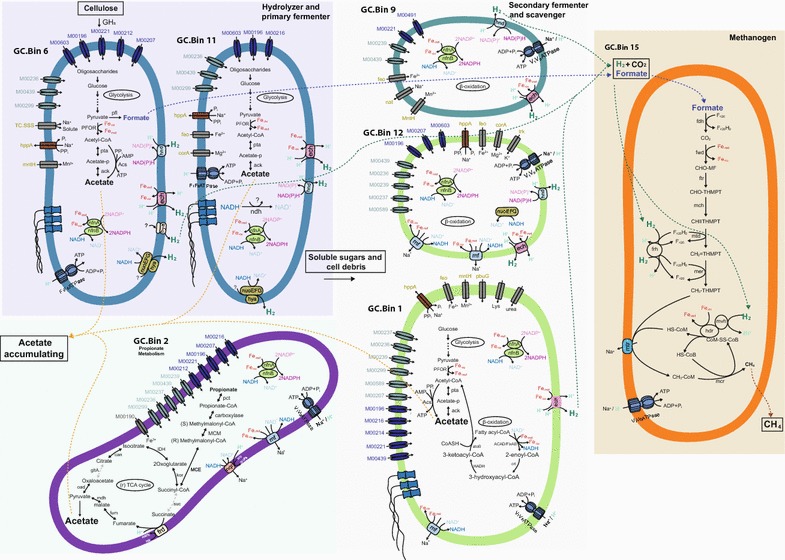
Schematic representation of the functional and ecological roles of the highly transcribed PGs in the GZ-C-35 enrichment culture. PGs were colored following the color code in Fig. 2. The schematics for the other cultures are shown in Additional file 8

In methanogenic systems, protons can be used as electron acceptors due to the low hydrogen partial pressure maintained by methanogens, increasing the energetic yield of anaerobic respiration [55]. In our enrichment communities, most of the highly transcribed fermenting bacteria likely coupled hydrogen production with re-oxidation of reduced cofactors to generate transmembrane proton gradients, recovering energy via membrane-bound proton driven ATP synthase (Fig. 5 and Additional file 8). SMF and membrane-bound sodium gradient driven ATP synthesis was also observed, with the reduced Fd-driven Rnf complex and the membrane-bound phosphatase-driven sodium pump being the most common means of establishing a sodium gradient (Figs. 3b, 5 and Additional file 8).

No obligate SAOBs were detected in the enrichment cultures, perhaps due to the use of serial dilutions during enrichment which may have diluted the slow-replicating SAOBs to extinction. All methanogens found in the enrichment cultures were hydrogenotrophs, leaving acetate generated from glycolysis to accumulate in the enrichment culture [24]. The highly transcribed fermenters were unlikely to be obligate syntrophic bacteria as they all possessed and transcribed sugar transport and fermentation pathways (Figs. 4, 5 and Additional file 8).

In all five enrichment cultures, only a small subset of the reconstructed PGs was highly transcribed. The meta-metabolisms of each enrichment culture were reconstructed from these highly transcribed core PGs (Fig. 5 and Additional file 8). In the 35 °C cellulose-amended methanogenesis enrichment culture GZ-C-35, the initial *C. cellulolyticum*-related hydrolyzers or primary fermenters (GC.Bin6 and GC.Bin11) hydrolyzed the substrates to oligosaccharides by attaching to the substrates via CBMs and/or cellulosomes, resulting in acetate as the major fermentation product and the transient production and complete consumption of formate (Fig. 5). Later, the secondary fermenting *C. leptum*-related organisms (GC.Bin12 and GC.Bin1) became functionally important and began scavenging fatty acids via β-oxidation (Fig. 5). Hydrogenotrophic methanogens scavenged fermented hydrogen and formate to keep the electron flow from cellulose to CH_4_ energetically feasible. A similar meta-metabolism and time course were observed in the SWH-C-35 culture, with *C. cellulolyticum*-related (SC.Bin7) and *Ruminococcus*-related (SC.Bin1) bacteria as hydrolyzers and primary fermenters, *C. leptum* (SC.Bin9, SC.Bin3) as secondary fermenters and potential fatty acid scavengers, and hydrogenotrophic *Methanobacteria* producing CH_4_ (Fig. 3a and Additional file 8a).

Similar meta-metabolism was inferred from the xylan-amended cultures, but the highly transcribed bacterial populations were phylogenetically distinct. *C. phytofermentans* (GX.Bin1)- and *C. saccharolyticum* (SX.Bin2)-related bacteria were the hydrolyzers and primary fermenters, while the *C. butyricum*-related bacteria (SX.Bin3 in SWH-X-35; GX.Bin3, GX.Bin25, GX.Bin4 and GX.Bin18 in GZ-X-35) were secondary fermenters and potential fatty acid scavengers (Fig. 3a and Additional file 8b, c). Although the bacterial communities for each substrate were distinct, the hydrogenotrophic *Methanobacteria* populations from all the 35 °C cultures were closely related (Fig. 3a).

In the 55 °C cellulose-amended culture, although a large fraction of the metatranscriptomic reads mapped to the unbinned contigs, the *E. harbinense*-related S5.Bin1 was identified as the primary hydrolyzer and the *T. thermosaccharolyticum*-related S5.Bin7 as the secondary fermenter and potential fatty acid scavenger (Fig. 3a and Additional file 8d).

## Conclusions

Reconstruction of the meta-metabolisms from the five cellulose- or xylan-digesting AD communities revealed that phylogenetically distinct microbial communities were assembled into similar meta-metabolic patterns. If the cultures had supported acetate consumption, we expect the meta-metabolisms to have been more complex, with acetoclastic methanogens and SAOBs playing a functional role. Although many PGs were reconstructed from each of the enrichment cultures, only a small subset of them was highly transcribed. The identified subset of highly transcribed functional players and the genes they express are candidates to be targeted as biomarkers for monitoring the performance of AD during treatment of cellulosic biomass. The meta-metabolic patterns observed in this study can provide guidance for the rational design of the AD microbiome to facilitate bioconversion of lignocellulosic substrates to methane.

## Additional files

**Additional file 1.** Sample description and sequencing summary of the (a) metagenome and (b) metatranscriptome samples.

**Additional file 2.** Summary statistics of de novo co-assembly and binning of metagenomes of the five enrichment cultures.

**Additional file 3.** Differential coverage binning results of the SWH-C-35 enrichment culture as an example (similar plots were generated for each culture). Differential coverage between any two of the four metagenomes collected at different time points from the same enrichment culture are shown respectively, with each axis representing the metagenome coverage of the contig at the labeled time point. A total of 28 high-quality PGs were extracted from the SWH-C-35 culture as shown in the figure.

**Additional file 4.** Detailed summary of the reconstructed PGs from the five enrichment cultures.

**Additional file 5.** Phylogenetic tree showing the placement of all the 107 reconstructed PGs across the five enrichment cultures. While 3737 reference genomes were used by PhyloPhlAn for alignment, only a subset of references was displayed. Blue diamonds indicate collapsed monophyletic clades with the number of reference genomes indicated in the brackets, black squares represent PGs from cultures amended with cellulose, and black triangles with xylan. Monophyletic clades of interest have been highlighted and bootstrap values (based on 100 iterations) are shown on internal nodes. The tree was midpoint-rooted. The scale bar indicates the evolutionary distance (substitution/site). The PGs are color-coded using the same scheme as in Fig. 3.

**Additional file 6.** Heatmap showing the transcriptional profiles of major CAZymes for the highly transcribed PGs based on log-transformed TPM values. The top 10 glycoside hydrolase families and top 5 families of other functional categories were plotted with other lower transcribed families grouped into the “Other” category. The grey color represents no detected transcription.

**Additional file 7.** Arrangement of the highly transcribed gene clusters associated with the (a) acyl-CoA dehydrogenase_FixAB complex and β-oxidation cycle, (b) Rnf complex, (c) NuoEFG hydrogenases, (d) Hnd hydrogenases, and (e) Ech and Hyp hydrogenases of the reconstructed PGs across the five enrichment cultures. Contig ID and PG ID are indicated on the left of each figure panel. Numbers on the Open Reading Frames (ORF) are the ORF ID and the numbers in brackets are sequence identities of each ORF against the corresponding ORF at the top of each panel.

**Additional file 8.** Schematic representation of the functional and ecological roles of the highly transcribed PGs in the (a) SWH-C-35, (b) GC-X-35, (c) SWH-X-35, and (d) SWH-C-55 enrichment cultures. PGs were colored following the color code in Fig. 2.
