# Supplementary figures and images for: Genome-centric metatranscriptomes and ecological roles of the active microbial populations during cellulosic biomass anaerobic digestion
Source: Biotechnol Biofuels. 2018 Apr 23;11:117. doi: 10.1186/s13068-018-1121-0 (PMC5911951; doi:10.1186/s13068-018-1121-0)

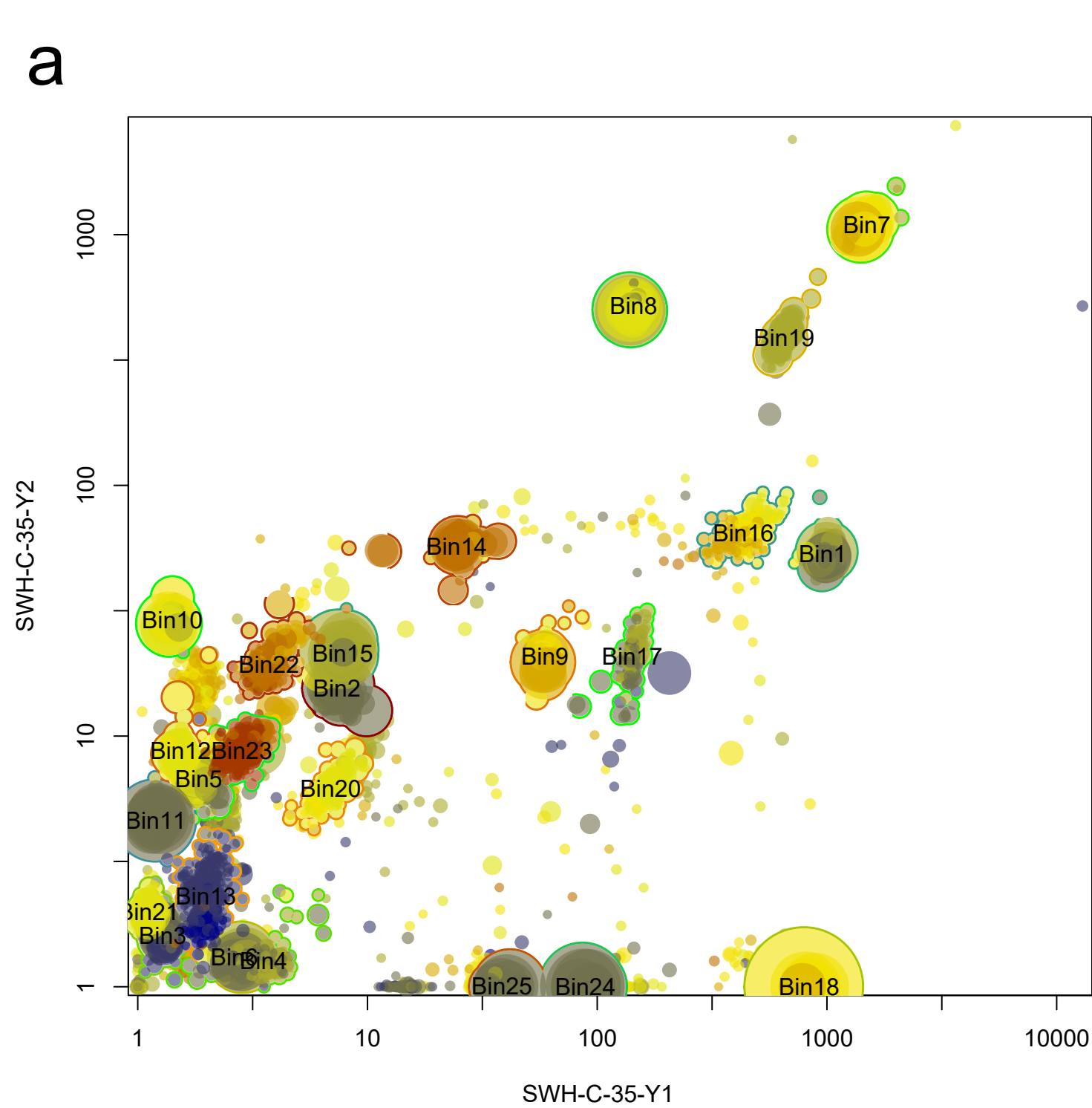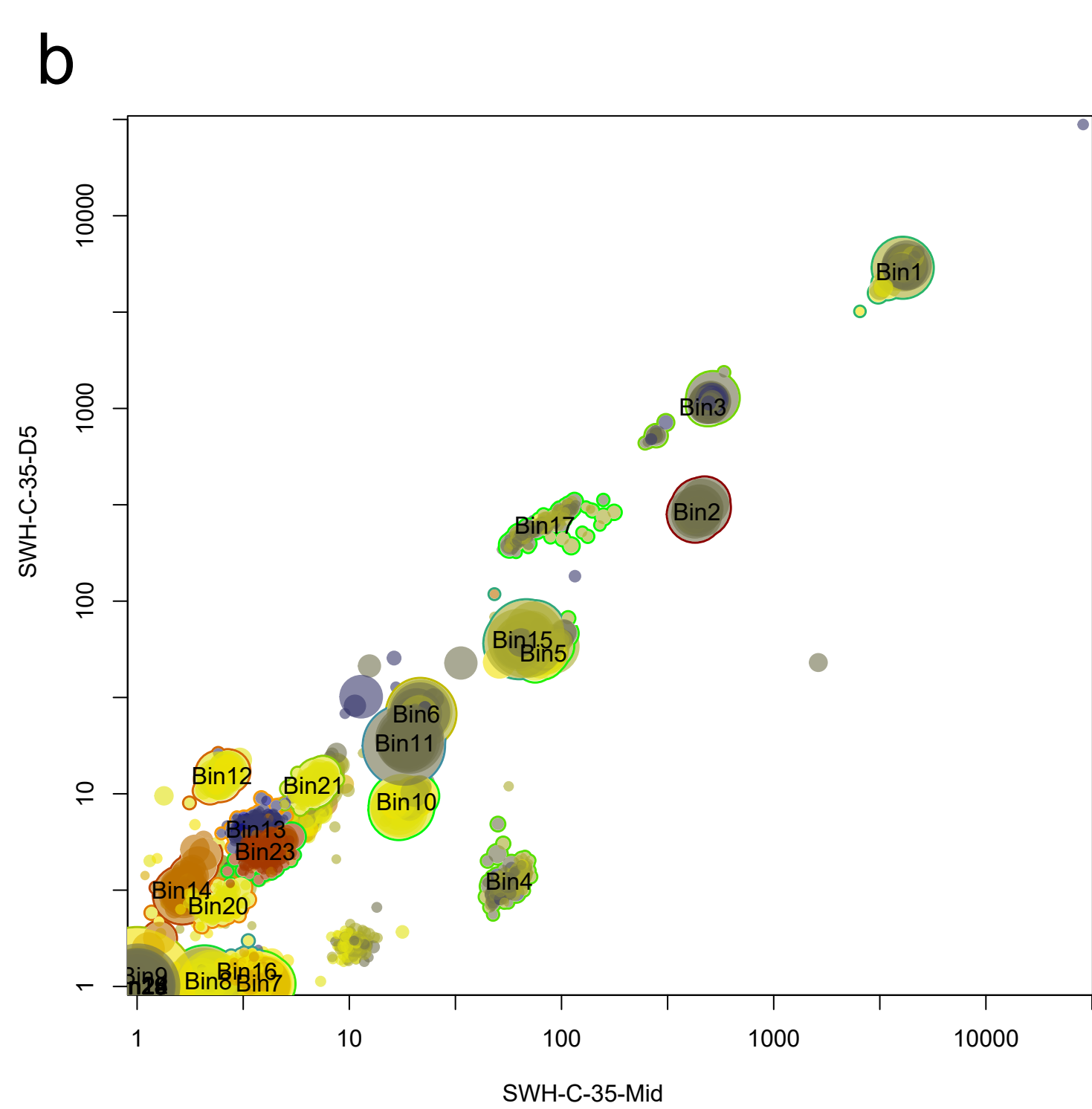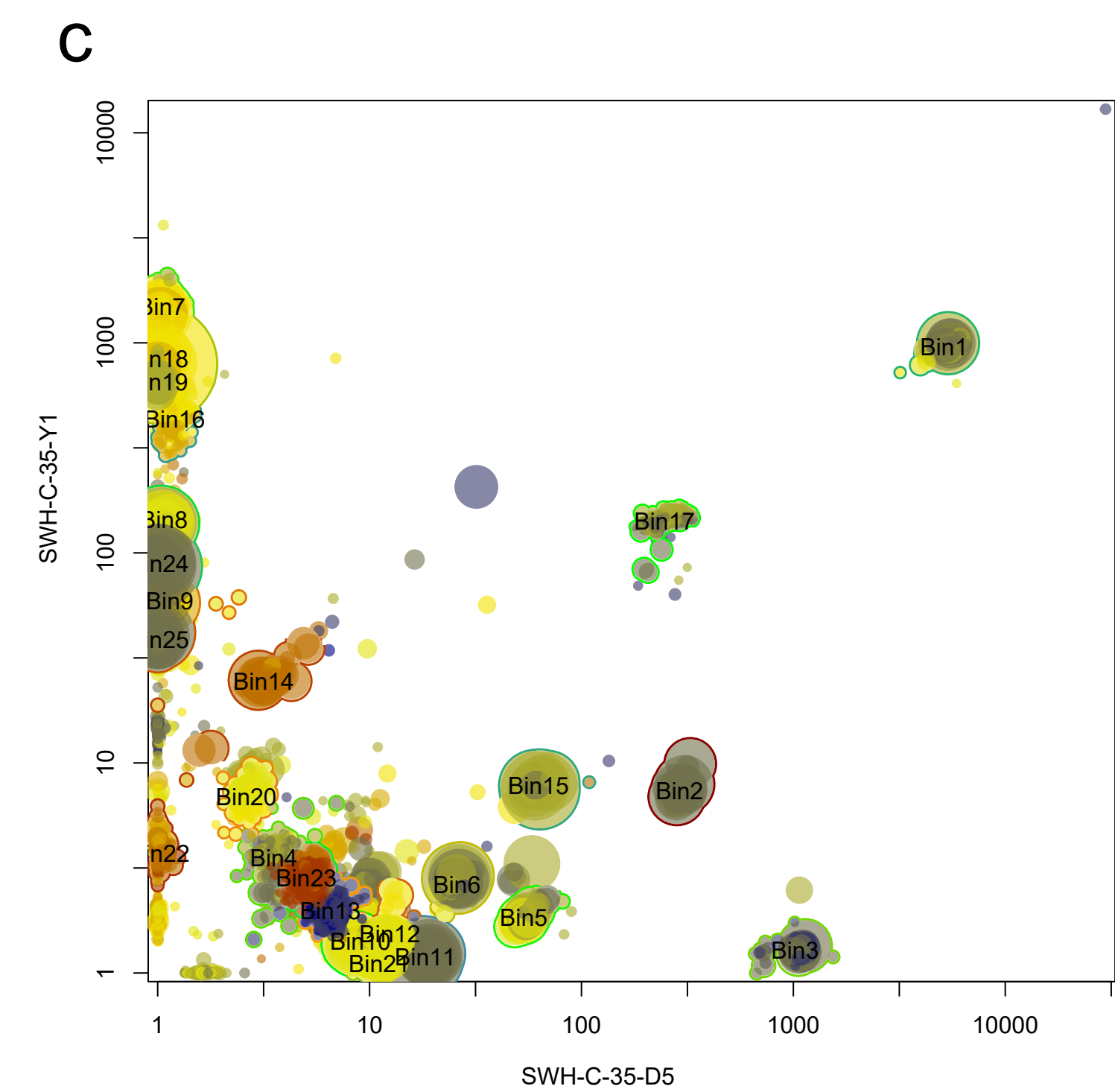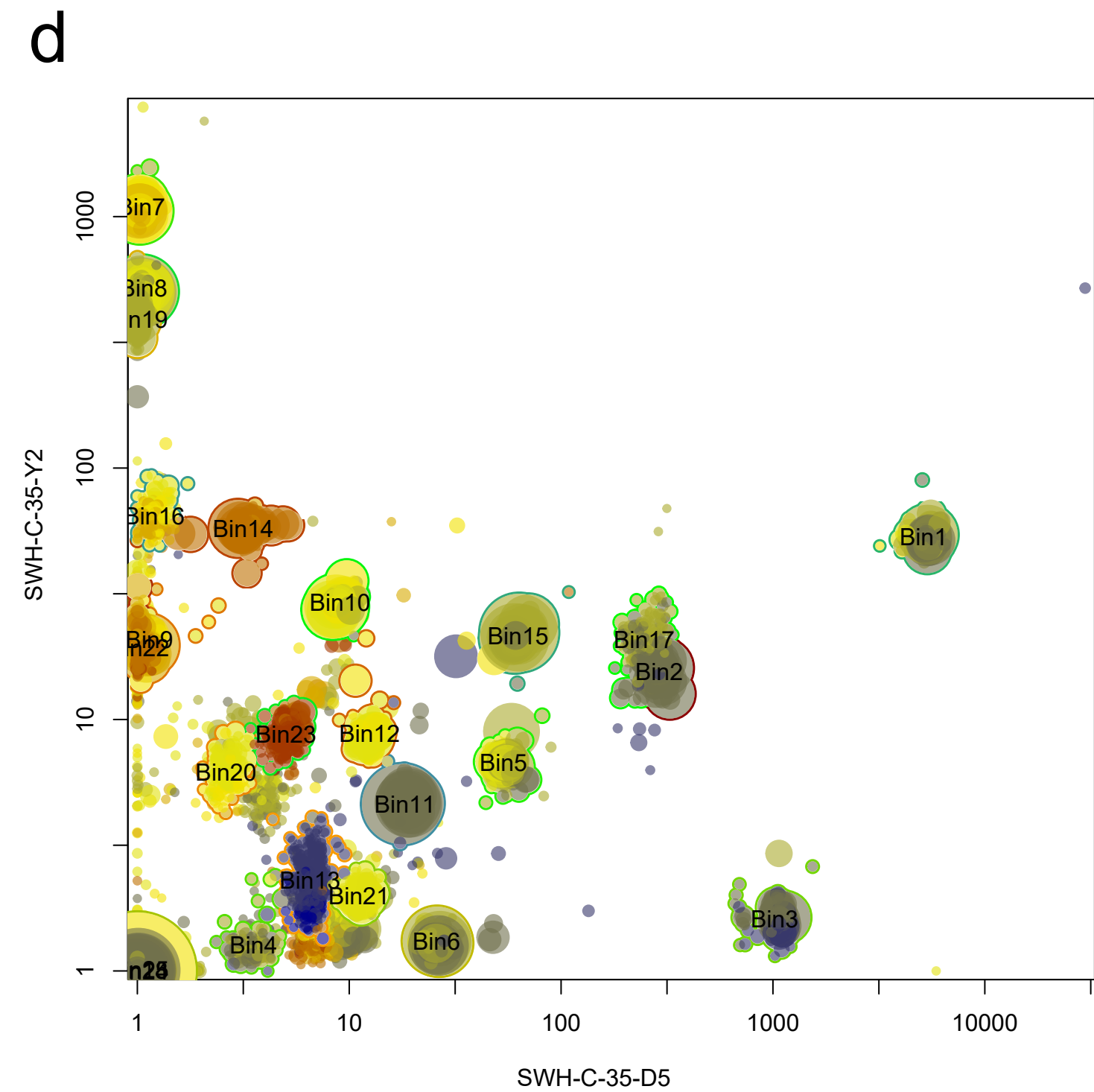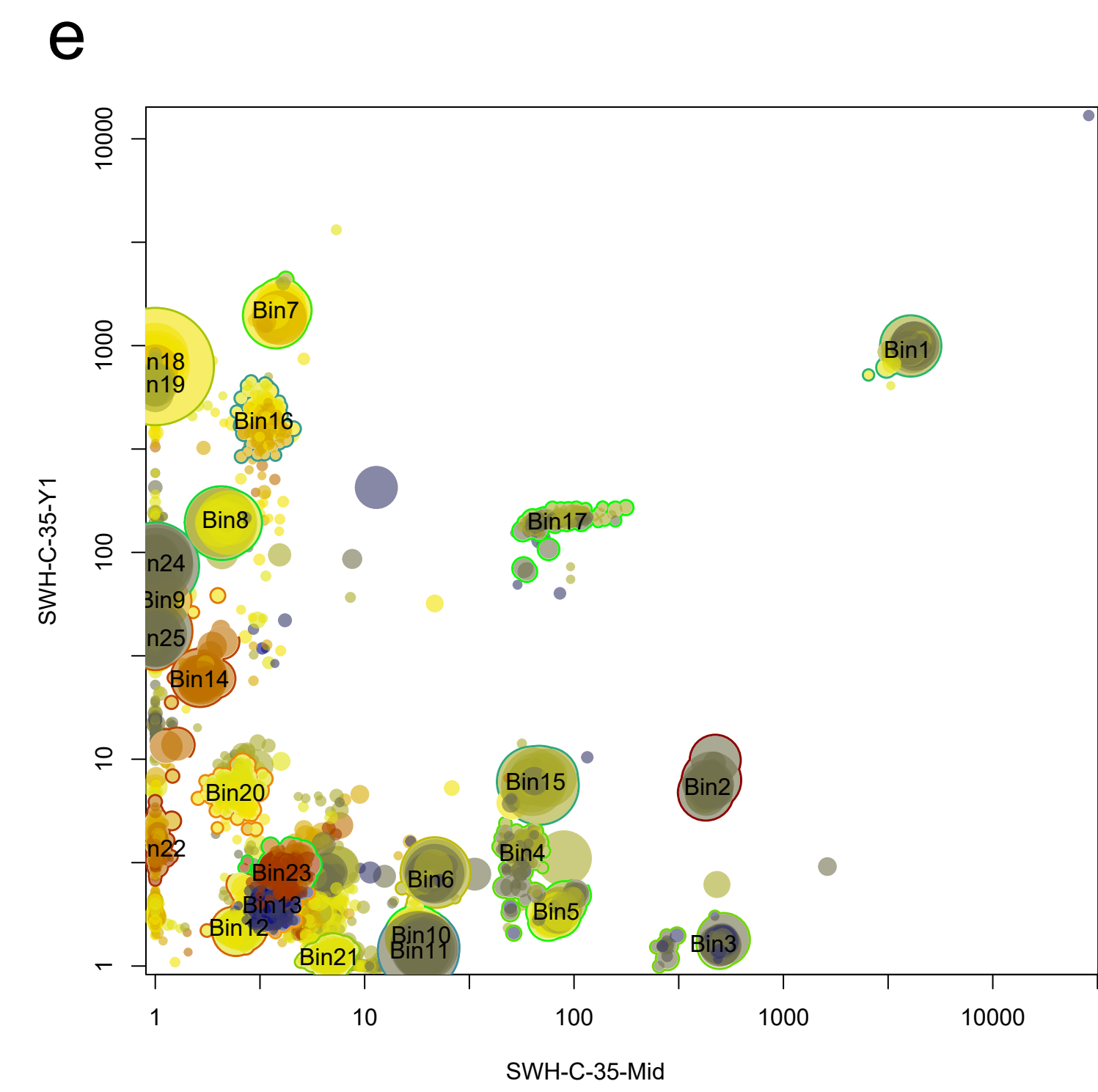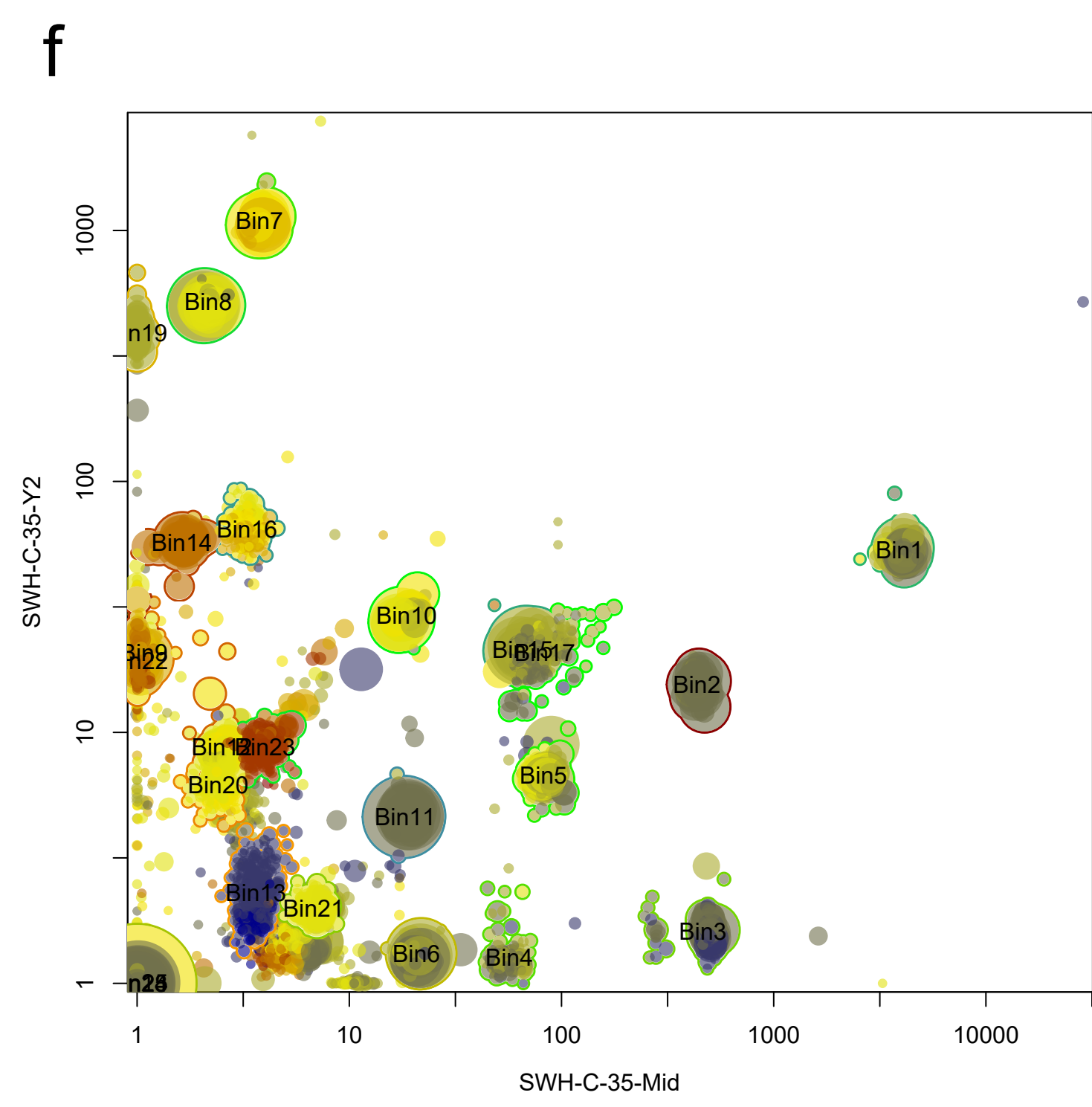

Supplement: Supplementary file 3 — Additional file 3. Differential coverage binning results of the SWH-C-35 enrichment culture as an example (similar plots were generated for each culture). Differential coverage between any two of the four metagenomes collected at different time points from the same enrichment culture are shown respectively, with each axis representing the metagenome coverage of the contig at the labeled time point. A total of 28 high-quality PGs were extracted from the SWH-C-35 culture as shown in the figure. [file 13068_2018_1121_MOESM3_ESM.pdf]

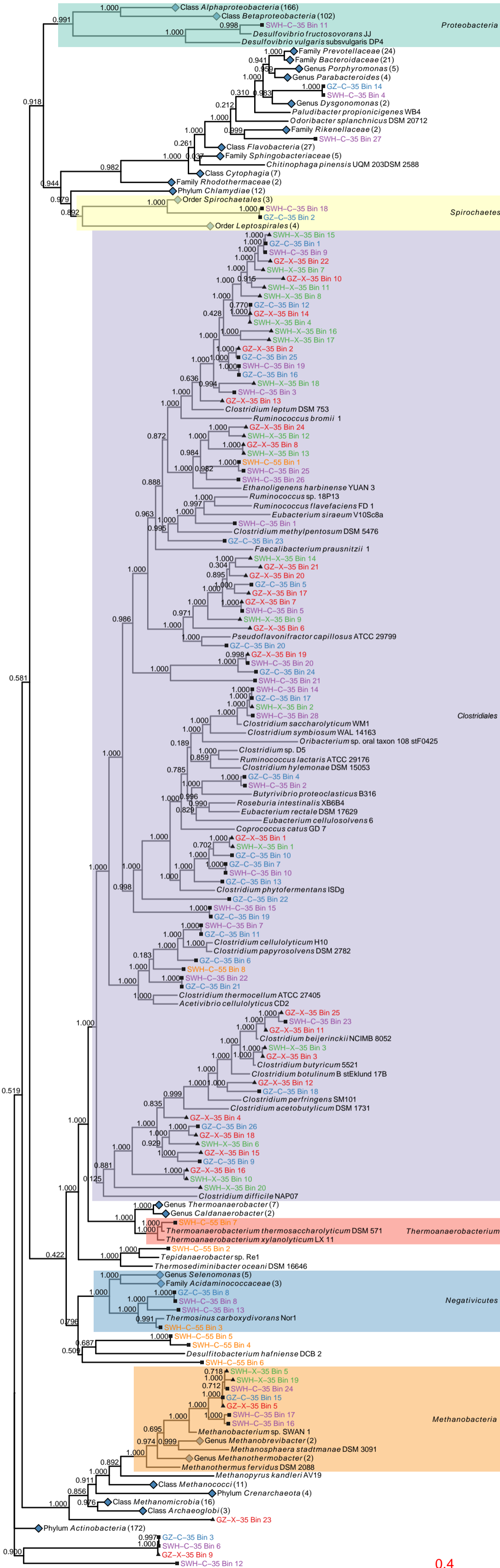

Supplement: Supplementary file 5 — Additional file 5. Phylogenetic tree showing the placement of all the 107 reconstructed PGs across the five enrichment cultures. While 3737 reference genomes were used by PhyloPhlAn for alignment, only a subset of references was displayed. Blue diamonds indicate collapsed monophyletic clades with the number of reference genomes indicated in the brackets, black squares represent PGs from cultures amended with cellulose, and black triangles with xylan. Monophyletic clades of interest have been highlighted and bootstrap values (based on 100 iterations) are shown on internal nodes. The tree was midpoint-rooted. The scale bar indicates the evolutionary distance (substitution/site). The PGs are color-coded using the same scheme as in Fig. 3. [file 13068_2018_1121_MOESM5_ESM.pdf]

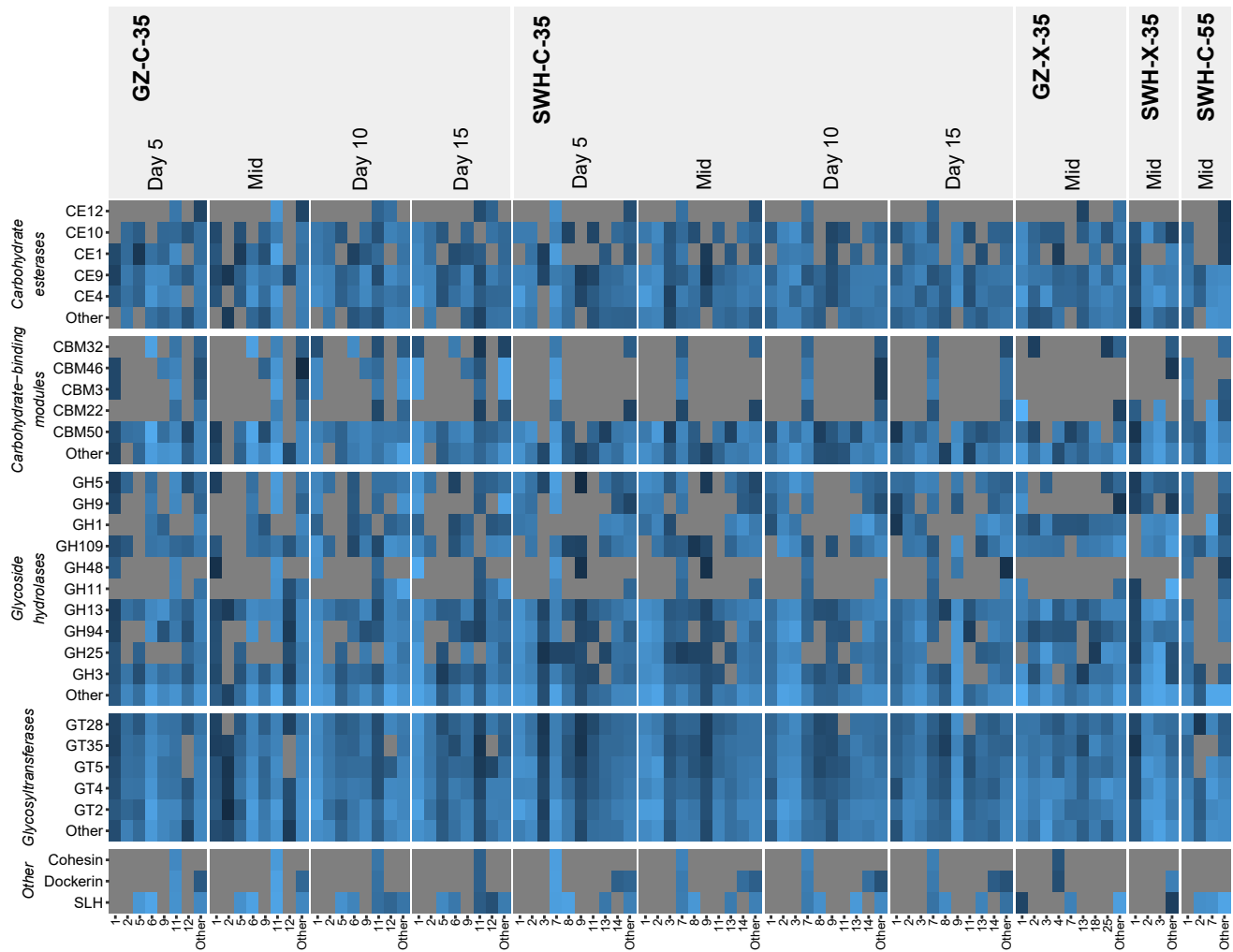

log(TPM)

0

5

Supplement: Supplementary file 6 — Additional file 6. Heatmap showing the transcriptional profiles of major CAZymes for the highly transcribed PGs based on log-transformed TPM values. The top 10 glycoside hydrolase families and top 5 families of other functional categories were plotted with other lower transcribed families grouped into the “Other” category. The grey color represents no detected transcription. [file 13068_2018_1121_MOESM6_ESM.pdf]

a

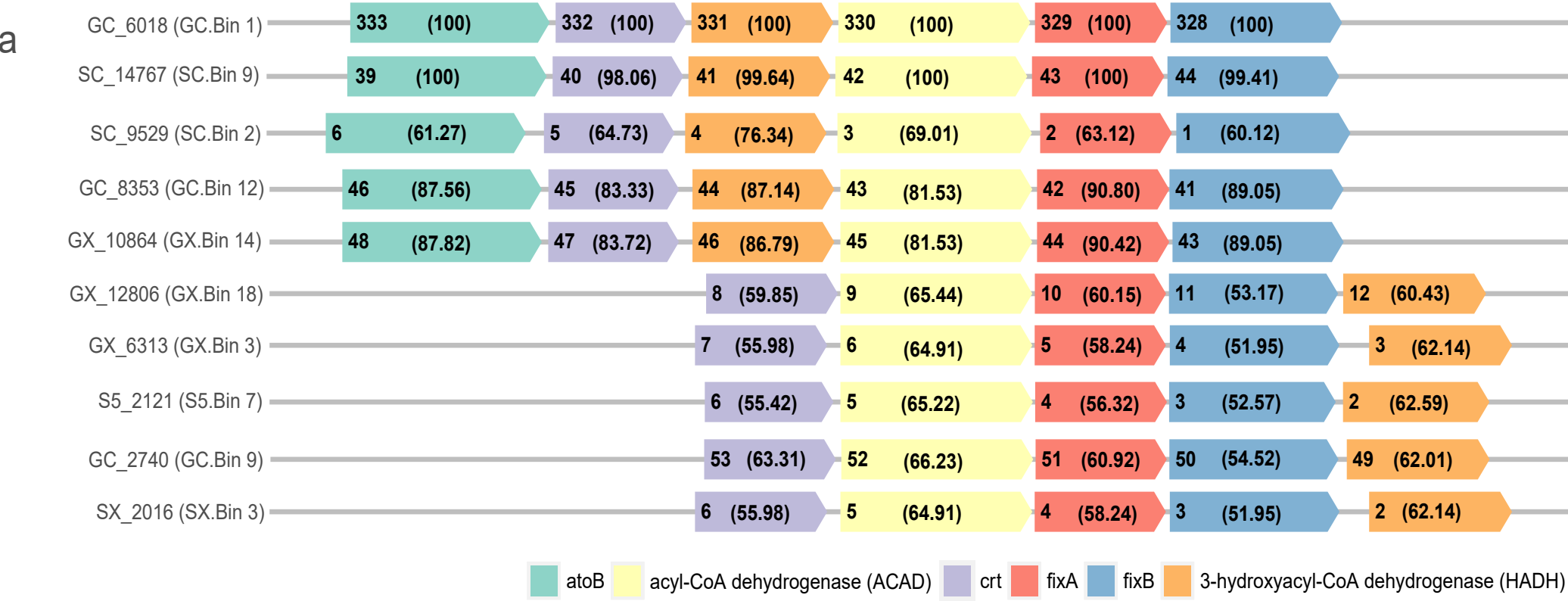

b

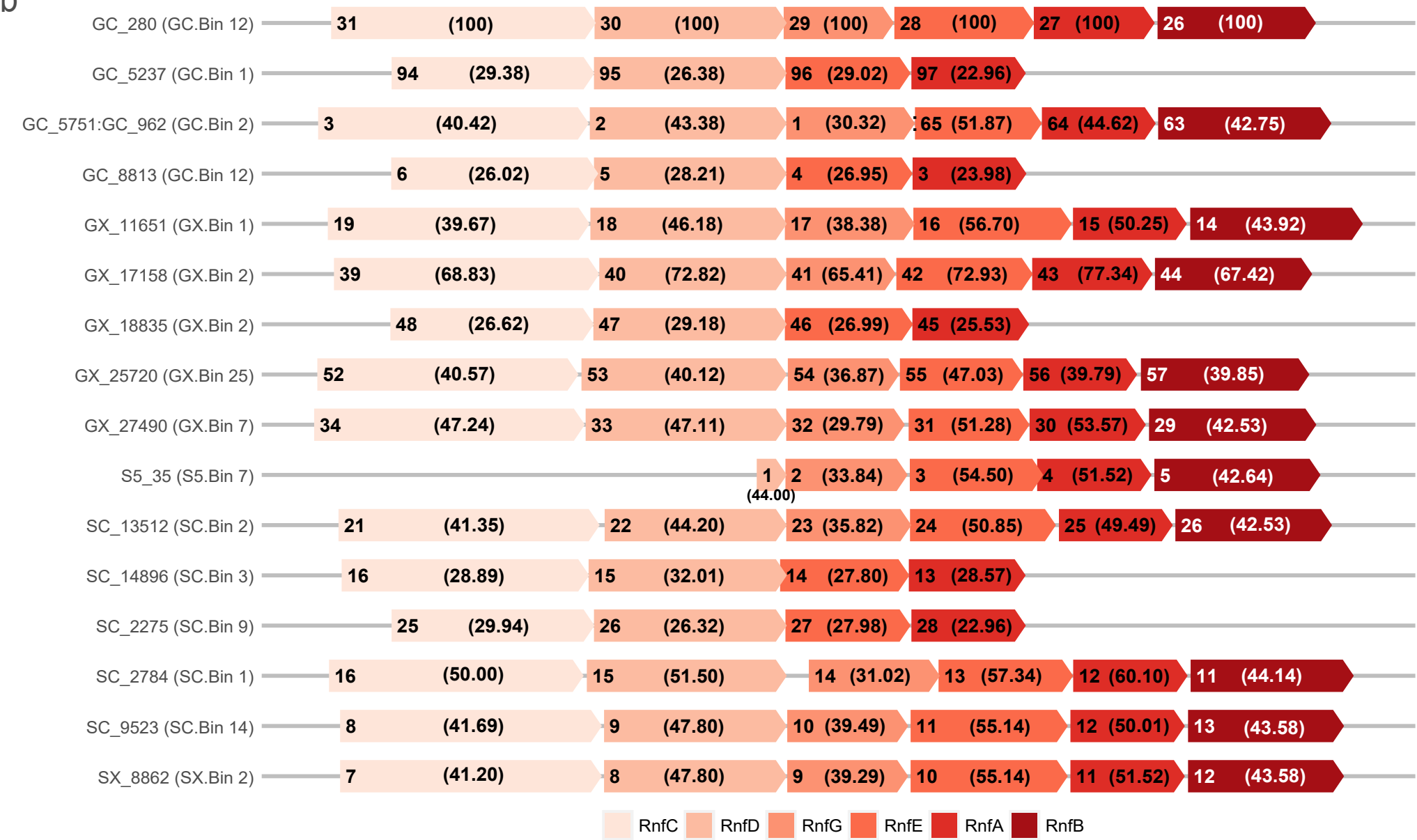

c

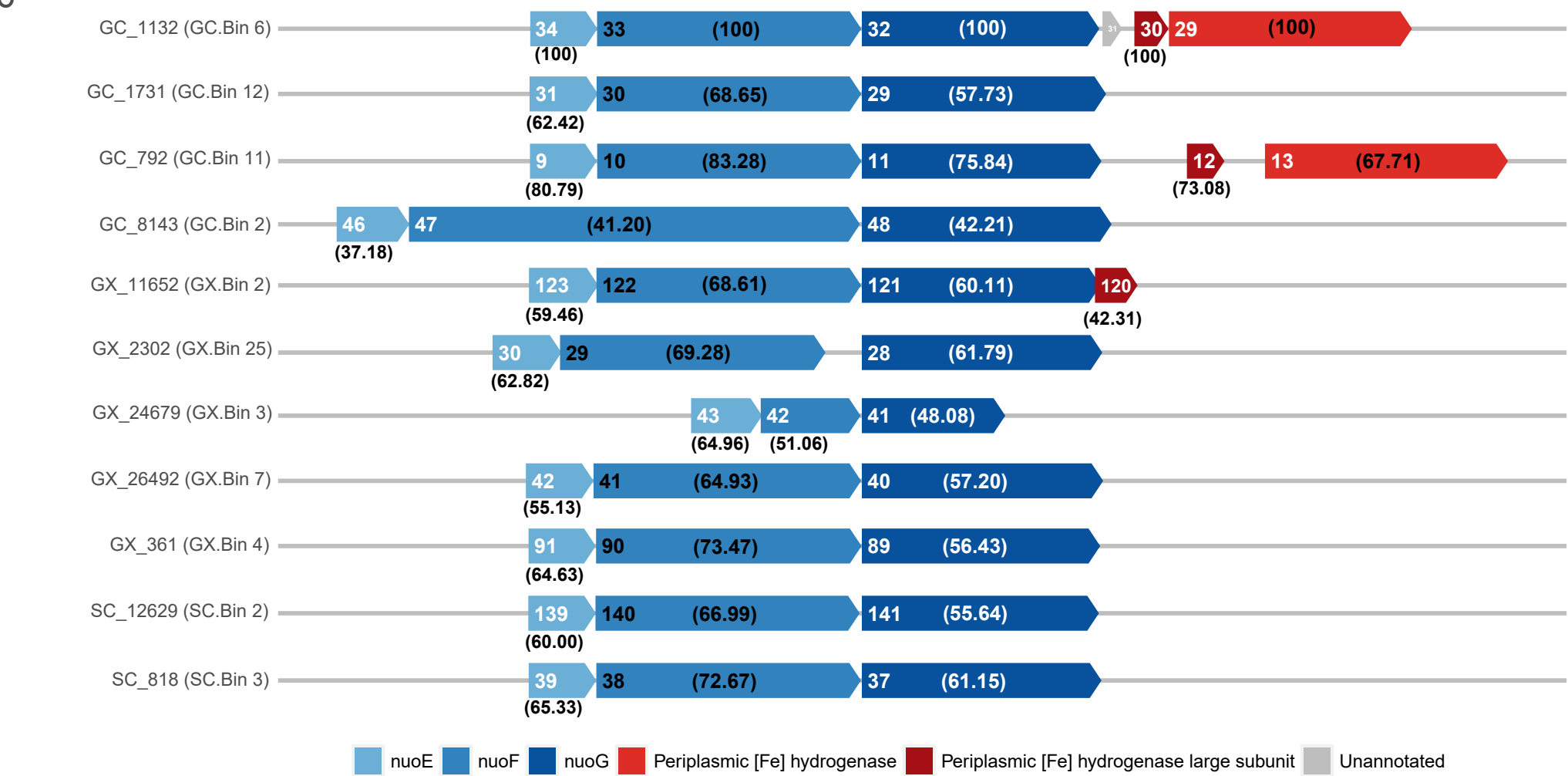

d

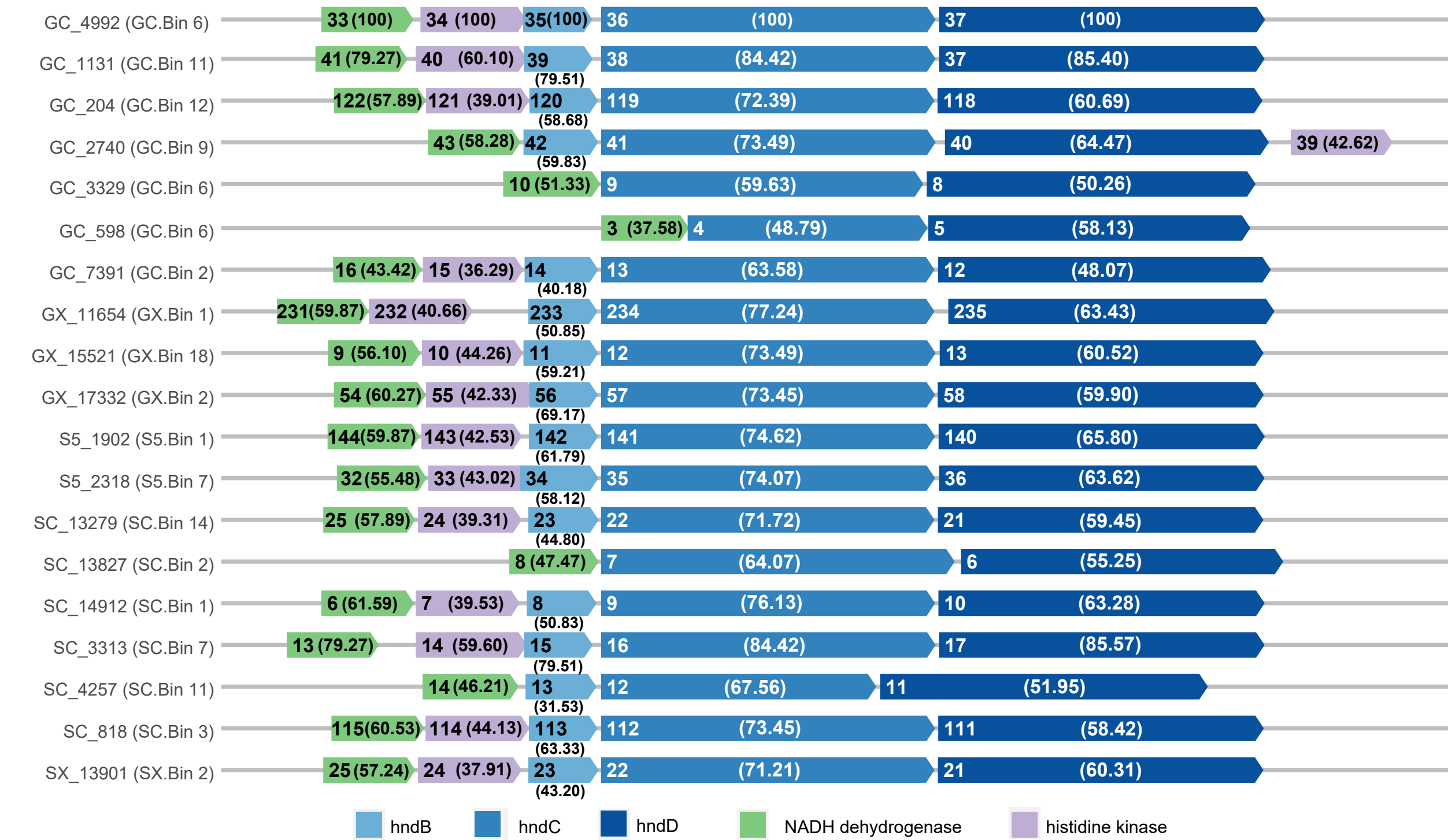

e

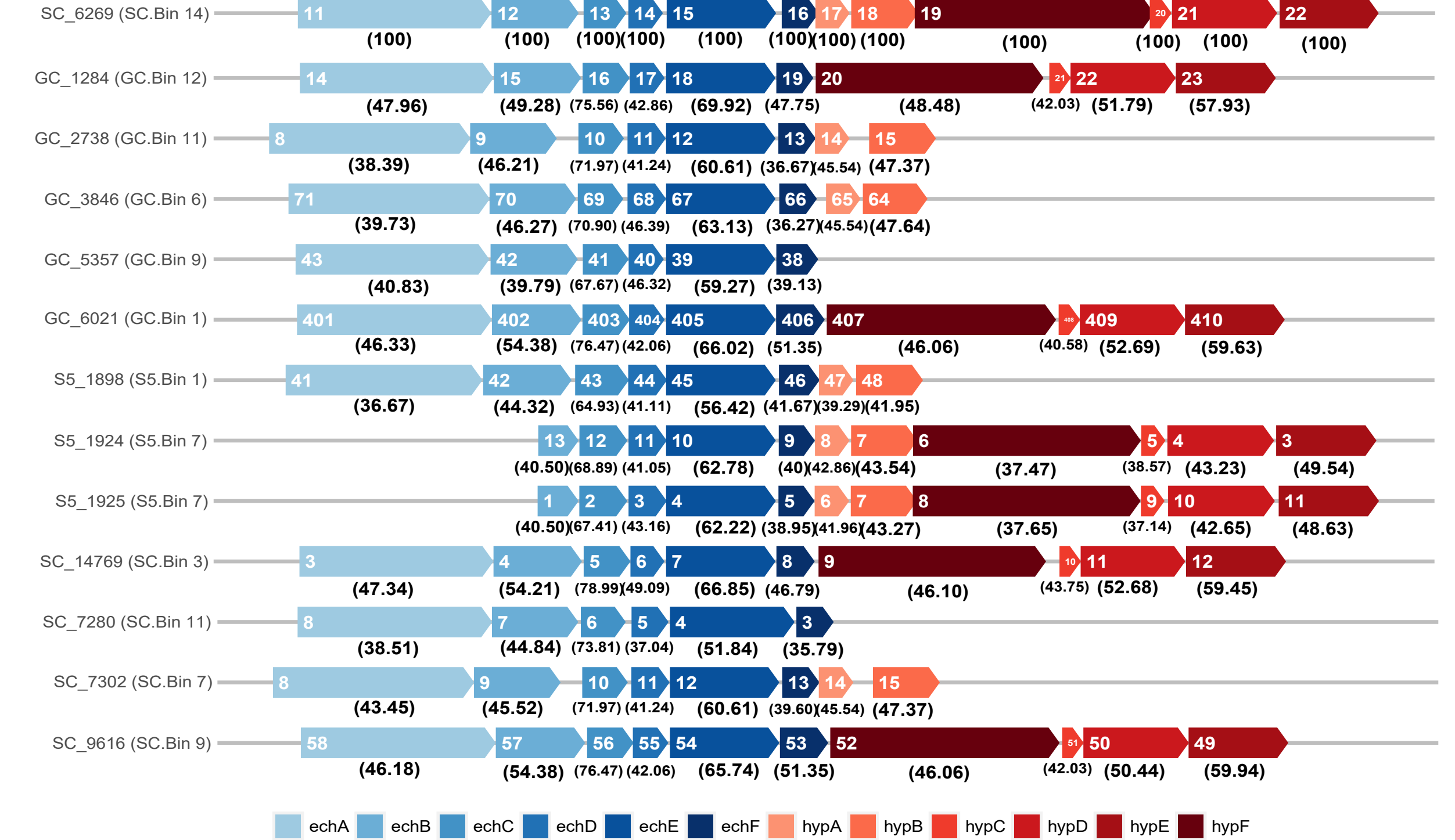

Supplement: Supplementary file 7 — Additional file 7. Arrangement of the highly transcribed gene clusters associated with the (a) acyl-CoA dehydrogenase_FixAB complex and β-oxidation cycle, (b) Rnf complex, (c) NuoEFG hydrogenases, (d) Hnd hydrogenases, and (e) Ech and Hyp hydrogenases of the reconstructed PGs across the five enrichment cultures. Contig ID and PG ID are indicated on the left of each figure panel. Numbers on the Open Reading Frames (ORF) are the ORF ID and the numbers in brackets are sequence identities of each ORF against the corresponding ORF at the top of each panel. [file 13068_2018_1121_MOESM7_ESM.pdf]
