# Supplementary material for: Genome-centric metatranscriptomes and ecological roles of the active microbial populations during cellulosic biomass anaerobic digestion
Source: Biotechnol Biofuels. 2018 Apr 23;11:117. doi: 10.1186/s13068-018-1121-0 (PMC5911951; doi:10.1186/s13068-018-1121-0)
Supplement: Supplementary file 8 — Additional file 8. Schematic representation of the functional and ecological roles of the highly transcribed PGs in the (a) SWH-C-35, (b) GC-X-35, (c) SWH-X-35, and (d) SWH-C-55 enrichment cultures. PGs were colored following the color code in Fig. 2. [file 13068_2018_1121_MOESM8_ESM.pdf]

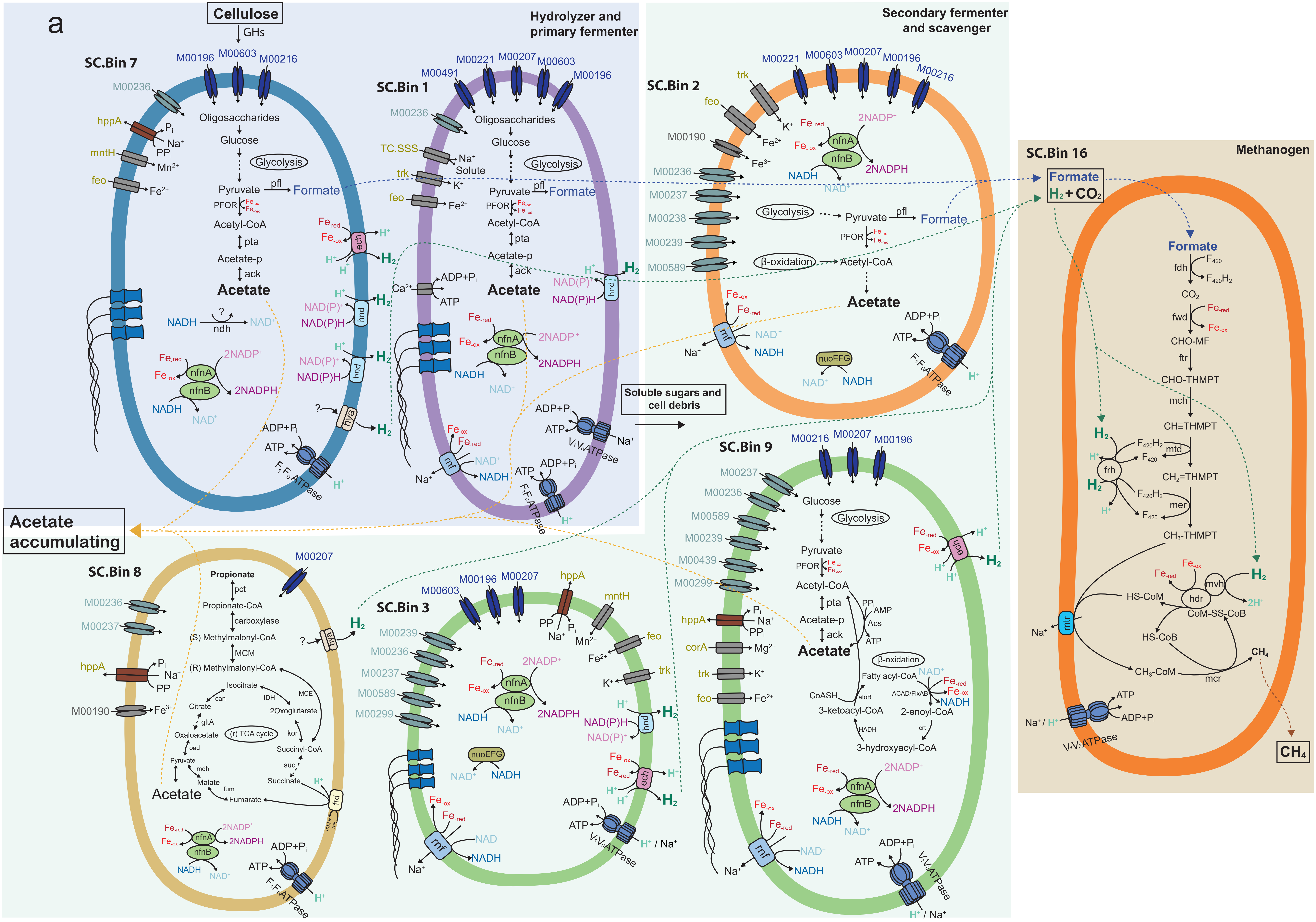
a
Cellulose
GHs
Hydrolyzer and primary fermenter
Secondary fermenter and scavenger
Methanogen
SC.Bin 7
SC.Bin 1
SC.Bin 2
SC.Bin 16
SC.Bin 8
SC.Bin 3
SC.Bin 9
Oligosaccharides
Glucose
Glycolysis
Pyruvate
pfl
Formate
PFOR
Acetyl-CoA
pta
Acetate-p
ack
Acetate
NADH
ndh
NAD+
nfnA
nfnB
2NADP+
2NADPH
ech
hnd
hya
mnf
nuoEFG
F1F0ATPase
V1V0ATPase
ADP+Pi
ATP
β-oxidation
Soluble sugars and cell debris
Acetate accumulating
Formate
H2 + CO2
fdh
CO2
fwd
CHO-MF
ftr
CHO-THMPT
mch
CH≡THMPT
mtd
CH2=THMPT
mer
CH3-THMPT
frh
mtr
HS-CoM
hdr
mvh
CoM-SS-CoB
HS-CoB
CH3-CoM
mcr
CH4
Propionate
pct
Propionate-CoA
carboxylase
(S) Methylmalonyl-CoA
MCM
(R) Methylmalonyl-CoA
Isocitrate
Citrate
2Oxoglutarate
Oxaloacetate
(r) TCA cycle
Succinyl-CoA
Malate
Fumarate
Succinate
frd
Fatty acyl-CoA
2-enoyl-CoA
3-hydroxyacyl-CoA
3-ketoacyl-CoA
CoASH
Acs
M00196
M00603
M00216
M00221
M00207
M00491
M00236
M00237
M00238
M00239
M00589
M00190
M00299
M00439
hppA
mntH
feo
trk
TC.SSS
corA

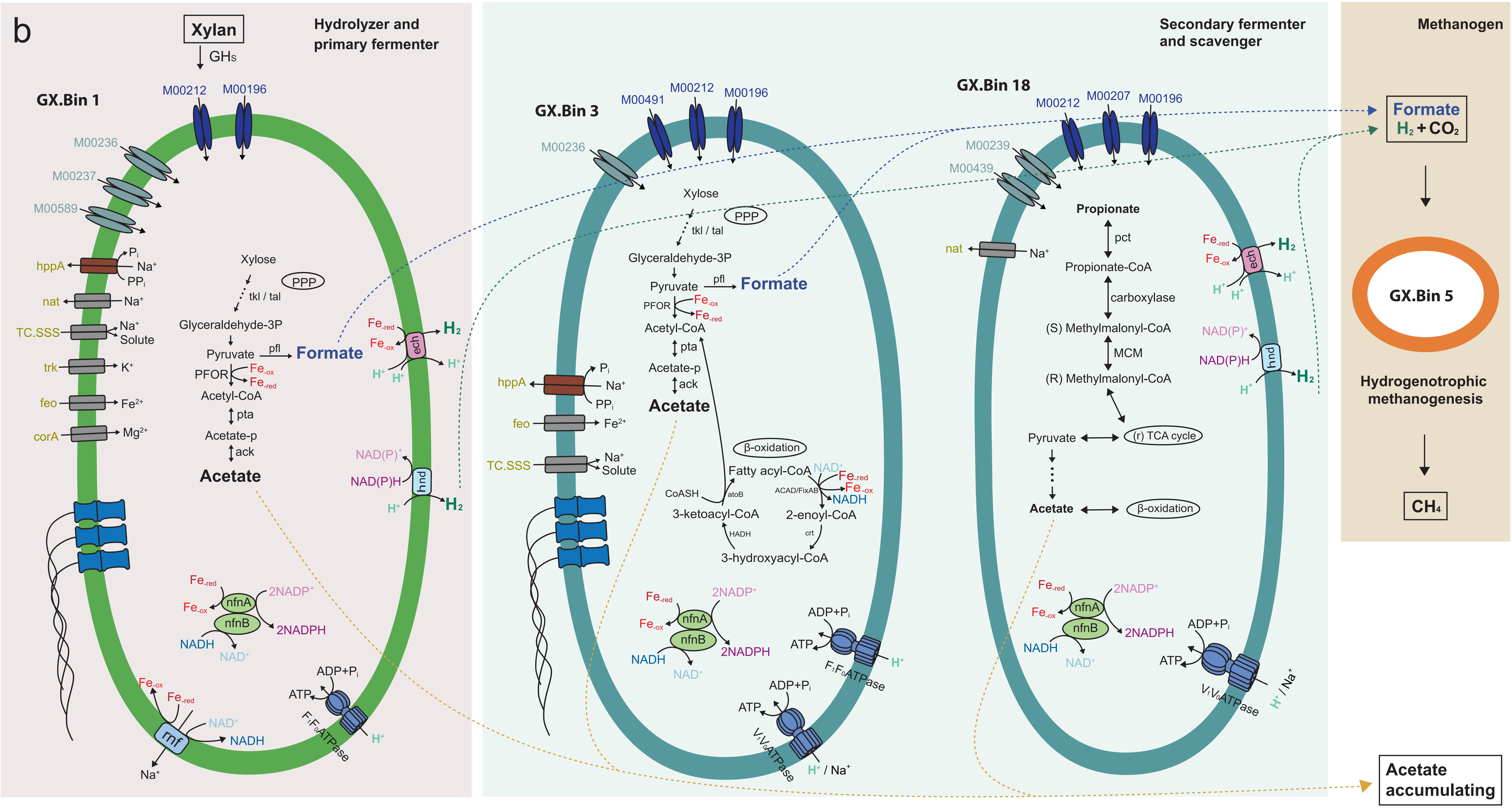
b
Xylan
GHs
Hydrolyzer and primary fermenter
GX.Bin 1
M00212
M00196
M00236
M00237
M00589
hppA
nat
TC.SSS
trk
feo
corA
Xylose
PPP
tkl / tal
Glyceraldehyde-3P
Pyruvate
pfl
Formate
PFOR
Acetyl-CoA
pta
Acetate-p
ack
Acetate
ech
H2
hnd
NAD(P)+
NAD(P)H
nfnA
nfnB
2NADP+
2NADPH
NADH
NAD+
rnf
ADP+Pi
ATP
F1F0ATPase
Secondary fermenter and scavenger
GX.Bin 3
M00491
β-oxidation
Fatty acyl-CoA
CoASH
atoB
ACAD/FixAB
3-ketoacyl-CoA
2-enoyl-CoA
HADH
crt
3-hydroxyacyl-CoA
V1V0ATPase
GX.Bin 18
M00207
M00239
M00439
Propionate
pct
Propionate-CoA
carboxylase
(S) Methylmalonyl-CoA
MCM
(R) Methylmalonyl-CoA
(r) TCA cycle
Methanogen
Formate
H2 + CO2
GX.Bin 5
Hydrogenotrophic methanogenesis
CH4
Acetate accumulating

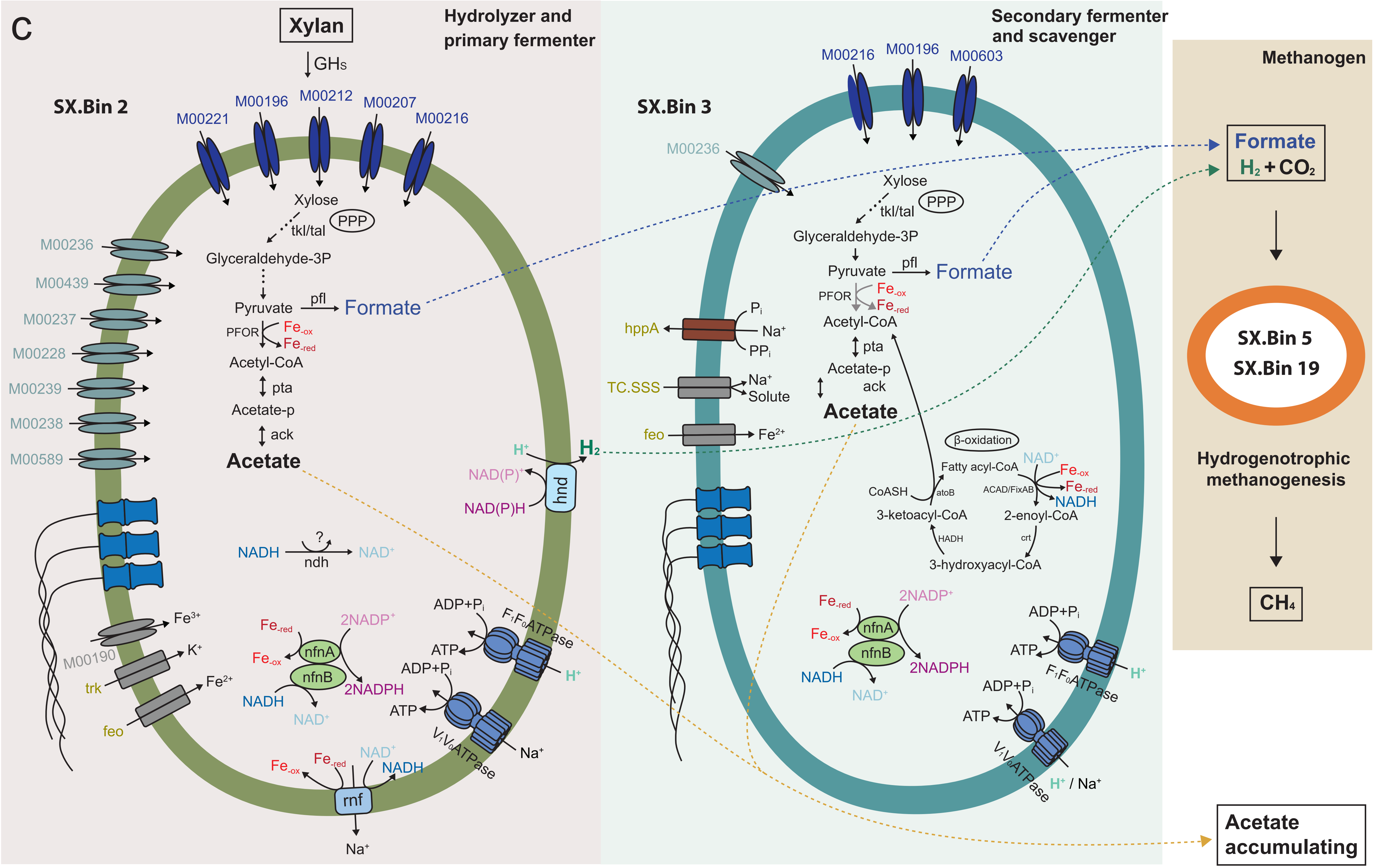
C
Xylan
GHs
Hydrolyzer and primary fermenter
SX.Bin 2
M00221
M00196
M00212
M00207
M00216
Xylose
tkl/tal
PPP
Glyceraldehyde-3P
Pyruvate
pfl
Formate
PFOR
Fe_{-ox}
Fe_{-red}
Acetyl-CoA
pta
Acetate-p
ack
Acetate
M00236
M00439
M00237
M00228
M00239
M00238
M00589
H^+
H_2
$NAD(P)^+$
NAD(P)H
hnd
NADH
?
ndh
NAD^+
Fe^{3+}
M00190
K^+
trk
Fe^{2+}
feo
Fe_{-red}
Fe_{-ox}
$2NADP^+$
nfnA
nfnB
2NADPH
NADH
NAD^+
$ADP+P_i$
ATP
$F_1F_0ATPase$
H^+
$ADP+P_i$
ATP
$V_1V_0ATPase$
Na^+
Fe_{-ox}
Fe_{-red}
NAD^+
NADH
rnf
Na^+
Secondary fermenter and scavenger
SX.Bin 3
M00216
M00196
M00603
M00236
Xylose
tkl/tal
PPP
Glyceraldehyde-3P
Pyruvate
pfl
Formate
PFOR
Fe_{-ox}
Fe_{-red}
Acetyl-CoA
pta
Acetate-p
ack
Acetate
P_i
hppA
Na^+
PP_i
TC.SSS
Na^+
Solute
feo
Fe^{2+}
β-oxidation
Fatty acyl-CoA
NAD^+
Fe_{-ox}
Fe_{-red}
ACAD/FixAB
NADH
CoASH
atoB
3-ketoacyl-CoA
2-enoyl-CoA
HADH
crt
3-hydroxyacyl-CoA
Fe_{-red}
$2NADP^+$
nfnA
Fe_{-ox}
nfnB
2NADPH
NADH
NAD^+
$ADP+P_i$
ATP
H^+
$F_1F_0ATPase$
$ADP+P_i$
ATP
$V_1V_0ATPase$
H^+ / Na^+
Methanogen
Formate
$H_2 + CO_2$
SX.Bin 5
SX.Bin 19
Hydrogenotrophic methanogenesis
CH_4
Acetate accumulating

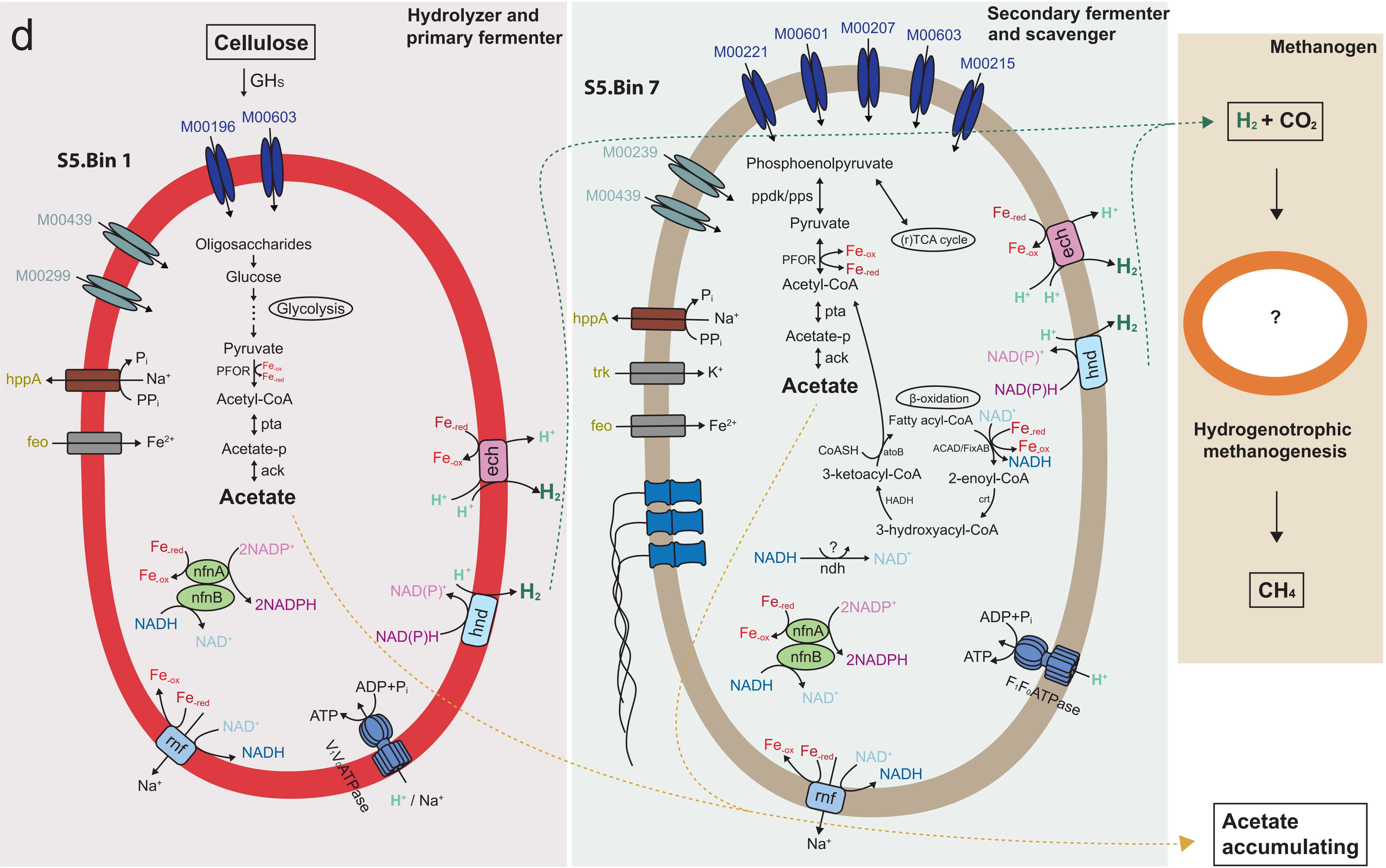

d
Cellulose
GHs
Hydrolyzer and primary fermenter
S5.Bin 1
M00196
M00603
M00439
M00299
Oligosaccharides
Glucose
Glycolysis
Pyruvate
PFOR
Acetyl-CoA
pta
Acetate-p
ack
Acetate
hppA
Pi
Na+
PPi
feo
Fe2+
ech
hnd
nfnA
nfnB
rnf
NADH
NAD+
2NADP+
2NADPH
NAD(P)+
NAD(P)H
H+
H2
ADP+Pi
ATP
V1V0ATPase
H+ / Na+
Secondary fermenter and scavenger
S5.Bin 7
M00221
M00601
M00207
M00603
M00215
M00239
M00439
Phosphoenolpyruvate
ppdk/pps
(r)TCA cycle
Acetyl-CoA
β-oxidation
Fatty acyl-CoA
CoASH
atoB
3-ketoacyl-CoA
ACAD/FixAB
2-enoyl-CoA
HADH
crt
3-hydroxyacyl-CoA
ndh
trk
K+
F1F0ATPase
Methanogen
H2 + CO2
?
Hydrogenotrophic methanogenesis
CH4
Acetate accumulating
